# Phytohormone-Mediated Regulation of Plant Cold Stress Tolerance: Signaling, Hormonal Crosstalk, and Translational Perspectives

**DOI:** 10.3390/ijms27094085

**Published:** 2026-05-02

**Authors:** Shafi Ullah, Mohammad Nurul Matin, Changxi Yin, Md. Atik Mas-ud, Atika Khan, Md. Shoffikul Islam, Irfanullah, Ijaz ul Haq

**Affiliations:** 1 College of Plant Science and Technology, Huazhong Agricultural University, Wuhan 430070, China; shafi@aup.edu.pk (S.U.); atkakhan7@gmail.com (A.K.); 2 Department of Biotechnology, Yeungnam University, Gyeongsan 38541, Republic of Korea; 3 National Key Laboratory of Crop Genetic Improvement, College of Plant Science and Technology, Huazhong Agricultural University, Wuhan 430070, China; yinchangxi@mail.hzau.edu.cn (C.Y.); atikgeb@gmail.com (M.A.M.-u.); 4 Department of Soil Science, University of Chittagong, Chattogram 4331, Bangladesh; msislam@cu.ac.bd; 5 College of life Science and Technology, Huazhong Agricultural University, Wuhan 430070, China; irfanullah.msbt652@gmail.com (I.), ijazulhaq1112@gmail.com (I.u.H.)

**Keywords:** phytohormones signaling, cold stress tolerance, crop resilience, signal transduction, gene expression, CRISPR/Cas9

## Abstract

Cold stress (CS) represents a major environmental factor that adversely affects plant growth, development, and productivity. To cope with low-temperature conditions, plants have evolved sophisticated mechanisms for CS perception and response, mediated through complex cellular signaling networks and physiological processes. Central to these adaptive responses are phytohormones, which function either independently or through synergistic and antagonistic interactions to fine-tune CS tolerance. This review synthesizes current knowledge on the roles of major classical phytohormones and signaling metabolites in regulating CS tolerance in plants. We first outline the molecular mechanisms involved in CS sensing and signal transduction, highlighting the roles of membrane-associated sensors, calcium signaling, and downstream transcriptional networks. Then, we discuss the contributions of key classical phytohormones, including auxin, abscisic acid, ethylene, salicylic acid, cytokinin, jasmonic acid, brassinosteroids, gibberellic acid, strigolactones, and signaling metabolites, including melatonin and gamma-aminobutyric acid, to CS tolerance, highlighting their individual and interacting roles in modulating gene expression regulation, antioxidant defense and physiological adaptations. We also discuss the crosstalk between these hormones, emphasizing the dynamic and often context-dependent nature of their interactions in response to CS. Furthermore, the review highlights recent advances in CRISPR/Cas9-based genome editing strategies targeting phytohormone biosynthesis, signaling, and response pathways to improve CS tolerance in plants. By integrating hormonal signaling, molecular regulation, and modern biotechnological tools, this review provides a comprehensive framework for understanding phytohormone-mediated CS adaptation and offers perspectives for developing climate-resilient crops through genetic and agronomic approaches.

## 1. Introduction

Plants are continuously exposed to a wide range of environmental stresses, including biotic (pathogen and pest-related) and abiotic (temperature, drought, salinity, and nutrient imbalance) stresses during their growth and development, posing a profound threat to global agriculture and sustainable food production. Climate change due to global warming is making this situation worse, demanding increased agricultural productivity to ensure food safety for the rapidly growing world population [1,2,3]. These stresses disrupt key physiological and biochemical processes such as photosynthesis, nutrient uptake, water balance, and cellular redox homeostasis, ultimately leading to reduced growth and yield [4,5,6]. For instance, drought and salinity stress induce osmotic imbalance and oxidative damage, while heat stress affects protein stability and membrane integrity. Increasing evidence suggests that plant responses to these stresses share common signaling components, including reactive oxygen species (ROS), calcium signaling, and phytohormone-mediated regulatory pathways. More specifically, plant adaptation to such environmental stresses involves highly complex mechanisms involving physiological adjustments, biochemical modulation, and molecular reprogramming that monitor the external environment and respond to external fluctuations, a process increasingly challenged by climate change [7,8,9]. Therefore, understanding abiotic stress responses in a broader context is essential for developing resilient crop systems under changing climatic conditions.

Among these common abiotic stresses, low-temperature stress, including chilling and freezing conditions, represents one of the most critical abiotic stresses, often limiting plant productivity, resulting in severe yield losses, which, under extreme conditions, may result in complete crop failure. There are two categories of low-temperature stresses: freezing and chilling, based on the temperatures and various physiological mechanisms that function in different temperature ranges [10]. Freezing stress occurs at temperatures below 0 °C and is characterized by ice crystal formation, cellular dehydration, and membrane rupture, and in contrast, chilling stress refers to exposure to low but non-freezing temperatures (typically 0–15 °C), leading to membrane rigidification, metabolic imbalance, and impaired enzyme activities [11,12,13]. Given the heterogeneity in stress conditions, both forms significantly impair plant growth, development, and productivity (Table 1). In this review, cold stress (CS) is used as an umbrella term encompassing both chilling stress and freezing stress unless otherwise specified. Where individual studies specifically address one condition, this distinction is explicitly stated throughout the manuscript.

Cold stress severely impacts plant growth and development by disrupting cellular homeostasis, membrane stability, photosynthesis, and metabolic processes, ultimately leading to significant yield and quality loss in many economically important crops, especially in temperate and subtropical areas [14,15]. Most crops are extremely sensitive to CS, which is associated with inhibited cell division, abated photosynthesis, and poor growth in general, even though some plant species have evolved increased tolerance to low temperatures [16,17]. The severity of cold-induced damage depends on multiple factors, including temperature intensity, duration of exposure, developmental stage, and species-specific sensitivity. With climate change driving increasingly unpredictable temperature fluctuations, understanding the molecular and physiological basis of CS responses and the underlying regulatory mechanisms of CS tolerance has become essential for developing climate-resilient varieties for safeguarding crop stability and food security.

**Figure 1:**
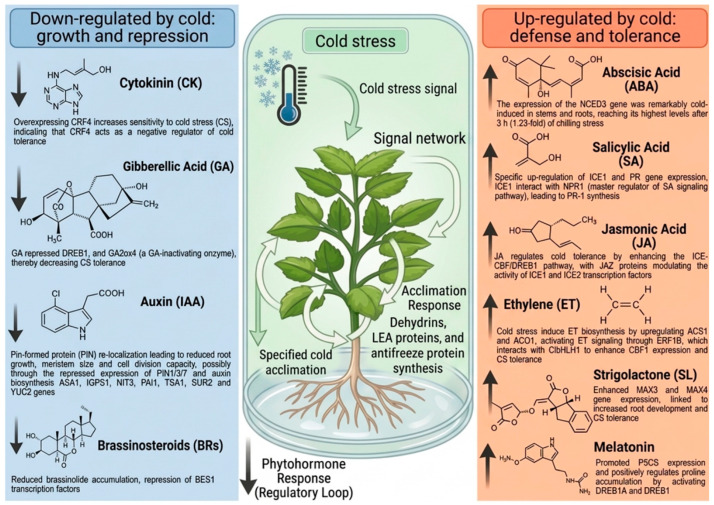
Overview of phytohormones regulating physiological responses to cold stress in plants. This figure summarizes the roles of key phytohormones in cold stress: abscisic acid, gibberellins, jasmonic acid, salicylic acid, cytokinin, auxin, ethylene, strigolactone, melatonin, and brassinosteroids, which regulate processes such as growth, defense, and acclimation under low-temperature conditions. On the left, downregulated hormones are shown, indicating a reduction in their levels under CS. On the right, upregulated hormones are depicted, highlighting an increase in their levels to mitigate the effects of low temperatures (figure created with BioRender.com).

Due to the unpredictable nature of global temperatures, molecular mechanisms of plant responses to CS are becoming increasingly important for the development of stress-resilient varieties. Plant responses to CS are mediated by intricate sensing, signaling, and adaptive systems. Plants have complex molecular mechanisms for detecting temperature changes and conveying this information to biochemical processes. Central to these processes is the regulation of phytohormone signaling networks, which act as the key regulators of stress adaptation and coordinate stress perception, signal transduction, and downstream gene expression (Figure 1). In addition to phytohormone-mediated signaling, transcription factors (TFs) and downstream functional genes play pivotal roles in orchestrating plant responses to cold and other abiotic stresses. Among these, the CBF/DREB (C-repeat binding factor/dehydration-responsive element binding) pathway represents a central regulatory module that activates a suite of cold-responsive (COR) genes, thereby enhancing freezing tolerance [18,19,20,21]. Other TF families, including MYB, NAC, WRKY, MYC, AP2/ERF (APETALA2/ethylene response factor), and bZIP, also contribute to stress adaptation by regulating gene expression associated with antioxidant defense, osmolyte accumulation, and cellular protection [18,22]. Several WRKY TFs from *Malus baccata* have been found to be largely activated in plant responses to stress, like *MbWRKY50,* which activated the expression of NCED, DREB, and CBF, thereby enhancing the resistance to CS and drought stress in tomato [23]; *MbWRKY33*, which increased high salinity stress tolerance in Arabidopsis [24]; *MbWRKY65*, which increased the tolerance to cold and drought in transgenic tomato [25]; and *MbWRKY5*, which increased drought and salt tolerance in transgenic tobacco [26]. Similarly, the grape WRKY TF VhWRKY44 enhanced cold and salt tolerance [27], VhMYB2 increased salinity and drought tolerance [28], and the clover WRKY TF TrWRKY41 improves cold tolerance [29] in Arabidopsis. The cold-inducible transcription factor BcWRKY53 positively regulates *BcCHLH* and *BcGUN4* and is actively involved in chlorophyll synthesis and the establishment of cold tolerance in Chinese cabbage [30]. These transcriptional regulators also function in close coordination with phytohormone signaling pathways, forming an integrated network that modulates plant stress responses at multiple levels.

The roles of phytohormones in regulating stress response, particularly CS, are well established, and include auxin [31,32,33], abscisic acid [34,35], gibberellins [36,37], jasmonic acid [38,39], ethylene [40], salicylic acid [41,42,43], cytokinin [44], strigolactones [45], melatonin [46], gamma-aminobutyric acid [47] and brassinosteroids [48,49]. Increasing evidence suggests that phytohormones not only act independently but also interact synergistically through a complex crosstalk network with signaling molecules that fine-tune plant responses to CS [44,50]. The large number of plant hormones suggests the existence of a highly interconnected hormonal signaling network that coordinates plant growth, development, and stress protection. Such networks enable plants to adapt efficiently to abiotic challenges while optimizing resource utilization. The major roles of phytohormones in alleviating CS are listed in Table 2.

Exogenous phytohormone application, as well as hormonal control, has demonstrated considerable potential in enhancing CS tolerance. Exogenous application of these hormones can be used to trigger the presence of inherent stress tolerance in plants, including activation of stress-related genes, generation of protective proteins, and cellular homeostasis. In addition, the emergence of genetic engineering instruments, including CRISPR/Cas9, has created novel opportunities for precise genome editing to improve cold tolerance [51]. Certain genes are now being targeted in gene editing to produce crops that are more resistant to CS, and this is one of the possible solutions to combat climate change. This review presents an in-depth summary of the molecular dynamics in CS detection and transduction, with special emphasis given to the interaction of the phytohormones that govern CS tolerance. The importance of different hormones to CS responses, the hormonal crosstalk underlying these responses, the prospects of exogenous hormone delivery, and genetic modification to increase tolerance in plants are also investigated in this review.

Although CS responses and phytohormone signaling have been extensively reviewed, most existing studies remain largely descriptive and compartmentalized, emphasizing single-hormone description and individual hormone pathways, particularly abscisic acid (ABA), or providing descriptive accounts of cold signaling without integrating the dynamic crosstalk among multiple hormones [52,53]. Reviews focusing on jasmonic acid, gibberellins, brassinosteroids, or auxin largely treat these pathways in isolation, thereby overlooking shared regulatory nodes and antagonistic or synergistic interactions that shape cold acclimation outcomes [50,54]. In addition, while CRISPR/Cas-based genome editing has rapidly emerged as a powerful tool for crop improvement, its application to systematically manipulate hormone crosstalk for cold tolerance remains fragmented and is rarely synthesized within cold-stress-focused reviews [55]. Consequently, there is a lack of crop-oriented frameworks that connect hormone-network regulation with actionable genome editing targets. Furthermore, the integration of multi-hormonal signaling networks under fluctuating environmental conditions is still poorly understood. This review addresses these gaps by integrating current knowledge of major phytohormone signaling under CS, highlighting shared regulatory nodes, and explicitly linking these nodes to CRISPR/Cas9-based genome editing strategies targeting hormonal pathways, with an emphasis on actionable gene targets and decision criteria for improving cold tolerance under different crop and environmental scenarios. By linking hormonal crosstalk with genome editing opportunities, this review offers a translational perspective that extends beyond prior descriptive accounts and provides a practical roadmap for climate-resilient crop development.

**Table 1:** Effects of cold stress (chilling and freezing) on growth and yield-related traits in major crops.

Plant Species	Stress Condition	Stress Duration	Reduction	References
Wheat	−6/−1/4 °C	2/4/6/days	5.3–14.9% yield reduction	[56]
Maize	13 °C	7 days	20% reduction in germination	[57]
Rice	3.4 °C below normal	Late April	12.7% yield reduction	[58]
Maize	8–14 °C		25% plant biomass reduction	[59]
Soybean	17/13 °C (day/night)	14 days	31.2% yield reduction	[60]
Wheat	−7 °C	20 days	8.0% yield reduction	[61]
Pepper	10/5 °C (day/night)	1 day	60% reduction in plant height	[62]
Canola	2–10 °C	1 day	3.3% yield reduction	[63]
Rice	10 °C	5 days	28.5% dry biomass and 32.9% plant height reduction	[64]
Fox grape	4 °C	4 days	19% reduction in plant height	[65]
Chickpea	13/7 °C (day/night)	4 days	27.8–34.6% reduction in pollen germination	[66]
Mung bean	10 °C	8 days	20–70% reduction in germination rate	[67]
Maize	4 °C	2 days	8.4% and 13.7% reduction in plant height and root length	[68]
Maize	4 °C	14 days	45% germination and 49.5% dry weight reduction	[69]
Pepper	10/8 °C (day/night)	30 days	7% reduction in dry biomass and 66% reduction in fresh biomass	[70]
Rice	4 °C	4 days	10% reduction in germination	[71]

This table provides an illustrative overview of representative studies across different crop species that report responses under varying temperature regimes, exposure durations, developmental stages, and measured endpoints, rather than a systematic or quantitative meta-analysis. Where possible, temperature ranges and response units have been standardized; otherwise, values are reported as described in the original studies.

**Table 2:** Roles of phytohormones in cold stress (chilling and freezing) responses in plants.

Hormone	Species	Treatment	Regulated Genes	Major Adaptive Response	Refs.
Abscisic acid (ABA)	*Arabidopsis thaliana*	Freezing (−6 to −8 °C); chilling (4 °C, 2–4 d)	*OST1/SnRK2.6, HOS1*, and *ICE1*	ABA-activated *SnRK2* signaling stabilizes *ICE1*, enhances *CBF* expression, and promotes osmoprotection and freezing tolerance.	[34]
Brassinosteroids (Br)	*Arabidopsis thaliana*	Freezing (−6 °C); chilling (4 °C)	*WRKY6*, *BZR1*, *SOC1*, *JMT*, *CBF* and *SAG21*	Modulation of cold-responsive (*COR*) and *CBF* expression. BR signaling via *BZR1* enhances freezing tolerance through *CBF*-dependent and independent pathways.	[48,49]
Gibberellic acid (GA)	*Arabidopsis thaliana*	Chilling (4 °C, 4 d)	*DELLA* and *GRF*	GA suppression leads to *DELLA* accumulation and interacts with *GRF* TF, restraining growth and promoting cold tolerance.	[36]
Jasmonic acid (JA)	*Arabidopsis thaliana*	Freezing (−9 °C, 1 h)	*DREB1*, *COI1*, *JAZ1/4*, and *ICE1/2*	JA signaling promotes freezing tolerance by relieving JAZ-mediated repression of ICE–CBF module.	[38]
Auxin	Cucumber (*Cucumis sativus*)	Chilling (5 °C, 120 h)	*YUCCA2*	Auxin homeostasis enhances chilling tolerance by reducing reactive oxygen species (ROS) accumulation and membrane damage while maintaining photosynthetic enzyme activities and efficiency.	[72]
Ethylene (ET)	*Arabidopsis thaliana*/Barley	Freezing (−1 °C); chilling	*EIN2*, *EIN3*, *CBFs*	ET acts as a negative regulator of freezing tolerance by repressing *CBF* expression while modulating antioxidant responses.	[73,74]
Cytokinin (CK)	*Arabidopsis thaliana*	Chilling (1 °C, 4 h)	*CRF2*, *CRF3*	CK response factors regulate root developmental plasticity and stress adaptation under CS.	[75]
Salicylic acid (SA)	*Arabidopsis thaliana*	Chilling (4 °C, 2 d)	*CPR5*, *BiP3*, *bZIP28*, *bZIP60*	SA improves membrane stability, photosynthetic efficiency, and stress-related UPR signaling during cold exposure.	[76,77]
Melatonin	Tomato (*Solanum lycopersicum*)	Chilling (4 °C, 24 h)	*SNAT* and *ASMT*	Enhances cold tolerance by improving photochemical efficiency, reducing ROS accumulation, and stabilizing membranes.	[78]
GABA	Wheat (*Triticum aestivum*)	Chilling (4 °C, 24–72 h)	*GAD*, *GABA-T*, *ALMTs*	GABA accumulation enhances antioxidant defense, regulates Ca^2+^ signaling, and improves membrane stability under CS.	[79,80]

The table summarizes representative studies highlighting hormone-regulated genes, signaling components, and adaptive responses under low-temperature stress. Cold stress is used as an umbrella term encompassing chilling (0–15 °C) and freezing (<0 °C) conditions. *OST1*; *OPEN STOMATA1*, GABA; gamma-aminobutyric acid.

## 2. Methods for Literature Collection

This review was conducted following a structured literature survey to identify peer-reviewed studies addressing phytohormone regulation, CS responses, and genome editing strategies in plants. Relevant literature was retrieved from Web of Science, Scopus, PubMed, and Google Scholar databases. The search covered publications from 2000 to 2026 (up to March), with particular emphasis on recent advances in hormone crosstalk and CRISPR/Cas-based approaches from the last 10 years. Search queries included combinations of keywords such as cold stress, low temperature, phytohormones, specific hormone naming, hormone crosstalk, cold tolerance, CRISPR/Cas9, genome editing, and crop improvement in conjunction with “plants.” Articles were selected based on the following criteria: (i) original research or authoritative reviews published in English in peer-reviewed journals; (ii) studies reporting molecular, genetic, or physiological mechanisms of hormone-mediated CS responses in plants; and (iii) research relevant to crops or model plant systems with translational significance. Studies focusing exclusively on non-cold abiotic stresses or lacking mechanistic insight and relevance to hormonal regulation were excluded. Priority was given to original research articles and high-quality reviews. Papers included in tables and figures were chosen based on their relevance to cross-hormone regulatory networks, experimental robustness, and contribution to identifying actionable genetic targets for cold tolerance. Final synthesis emphasizes consistency across studies and integrates evidence to highlight regulatory nodes and genome editing opportunities.

## 3. Cold Stress Sensing and Signal Transduction

**Figure 2:**
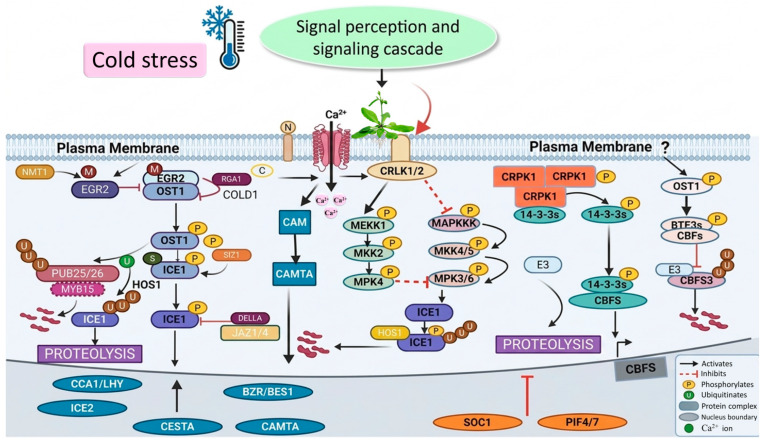
Endogenous pathways of CS sensing and signal transduction in plants. Cold signal detection is carried out by the plasma membrane, prompting its increased rigidity through lipid composition changes. The cold sensor (COLD1/RGA1) and other components activate Ca^2+^ channels, elevate cytosolic Ca^2+^ levels, and activate plasma membrane protein CRLK1/2. This triggers the MEKK1-MKK2-MPK4 signaling cascade and inhibits MAP KINASE 3/6. MAPK signaling converges on the ICE1–CBF–COR regulatory pathway. MPK3/6-mediated phosphorylation of ICE1 promotes its ubiquitination and degradation by *HOS1*, limiting ICE1 binding to CBF promoters. In contrast, *OST1* phosphorylates ICE1, enhancing its transcriptional activity and stability. ICE1 stability is further promoted by the E3 SUMO ligase SIZ1, which counteracts *HOS1*-dependent degradation. During cold stress, *OST1* also phosphorylates BTF3, contributing to CBF stabilization. Additionally, 14-3-3 proteins, phosphorylated by CRPK1, translocate to the nucleus to induce CBF degradation, regulating CS response. CS inhibits the myristoylation of EGR2, disrupting EGR2–*OST1* interaction and activating *OST1*. Activated *OST1* phosphorylates ICE1, preventing its degradation by *HOS1*, enhancing its stability and promoter binding ability to CBFs. COLD1, CHILLING-TOLERANCE DIVERGENCE 1; RGA1, heterotrimeric G-protein α subunit; CRLK1/2, CaM-regulated receptor-like kinase; CRPK1, COLD-RESPONSIVE PROTEIN KINASE 1; MEKK1-MKK2-MPK4, MAPK KINASE KINASE 1–MAP KINASE KINASE 2–MAP KINASE KINASE 4; ICE1, inducer of CBF expression 1; CBF, C-repeat (CRT) binding factor; COR, cold-regulated; *HOS1*, HIGH EXPRESSION OF OSMOTICALLY RESPONSIVE GENE 1; *OST1*, OPEN STOMATA 1; EGR2, EARLY GROWTH RESPONSE 2; SUMO, SMALL UBIQUITIN-LIKE MODIFIER, SIZ1, SUMO E3 ligase 1; BTF3, basic transcription factor 3. The ‘?’ mark in the plasma membrane refers to a signaling pathway where the exact initial sensor or the precise mechanism of various protein kinases and transcription factors that mediate the plant’s response to low-temperature environments is still being researched. Figure created with BioRender.com.

Cold signaling in plants has been the subject of considerable research; however, the precise mechanisms by which plants detect low temperatures are still not fully clear. Cold exposure has been shown to increase the production of secondary messengers, including calcium ions (Ca^2+^), which are recognized by specific sensors, often referred to as “secondary sensors,” to activate the plant’s adaptive responses to CS. As a widespread and crucial secondary messenger, Ca^2+^ plays a key role in regulating a variety of physiological processes and coordinating the signaling networks that mediate responses to abiotic stress (Figure 2). Protein receptors embedded in the plasma membrane detect low temperatures and trigger changes in membrane structure. This, in turn, results in the movement of calcium ions in the cytosol and the transmission of signals to other cell compartments [81]. Cold stress responses have been reported to be associated with plasma membrane rigidification and cytoskeleton rearrangements. This response is mediated by microtubules and microfilaments, which play a role in opening Ca^2+^ channels. Furthermore, in *Vicia faba*, the entry of Ca^2+^ into sieve elements in response to cold shock is regulated by the cytoskeleton, resulting in sieve plate blockage. Several cellular processes are modulated by complexes involving Ca^2+^ and calcium sensors, including ion transport, gene expression regulation, metabolic activities, and post-translational modifications [82]. In rice, the protein complex COLD1/RGA1, located on the plasma membrane, plays a key role in detecting low-temperature stress. Upon sensing cold, COLD1 activates the G-protein α subunit, which interacts with Ca^2+^ and triggers the downstream DREB (dehydration-responsive element binding) signaling pathway, thereby enhancing cold tolerance in the plant [83].

Low-temperature perception in Arabidopsis entails the opening of mechanosensitive Ca^2+^ channels, namely mid1 complementation activity (MCA1), mid2 complementation activity (MCA2), and a Ca^2+^ permeable transporter called *ANNEXIN1* (*AtANN1*). These are channels that mediate Ca^2+^ influx under low temperatures and trigger the ensuing signal transduction pathways [84]. Signal propagation is subsequently mediated by Ca^2+^ sensors, namely CBLs, CaM, CDPKs (calcium-dependent protein kinase), and CIPKs (calcium-interacting protein kinase). Cytosolic calcium regulation alleviates the adverse effects of Ca^2+^-induced ROS production, which can otherwise impair cellular function [85]. The increase in calcium in plants in response to CS is regulated by Calcineurin B-like proteins through CIPK, the serine–threonine protein kinase. In Arabidopsis, CBL1 and CIPK7 enhance freezing tolerance by activating COR (cold-regulated) gene expression [86]. CBLs and CIPKs are plant-specific regulators, and their involvement is triggered by CS. Genome-wide analyses in cassava revealed tissue-specific expression of CBL and CIPK family members under CS, while CBL5 and CIPK14 were expressed in the leaves only [87]. *OsCDPK27* is a calcium ion sensor that is a useful regulator of cold tolerance in rice. The cold signaling is activated through nitric oxide (NO), ROS, and MAPK-mediated pathways [88]. *OsCDPK17* is crucial in the plant’s response to CS, where it facilitates the opening of membrane channels and contributes to the breakdown of sugars [89].

Plants possess diverse non-selective cation conducting (NSCC) channels, forming cyclic nucleotide-gated channels (CNGCs) and glutamate receptors (GLRs) that are located in selectively permeable membranes. Specifically, CNGCs maintain cytosolic Ca^2+^ homeostasis without regulating other monovalent ions [90]. In rice, CNGCs are classified into four groups (I–IV), with groups I–III upregulated and group IV downregulated under CS. Moreover, in order to mitigate CS injury in rice, the expression of *OsCNGC6* is elevated, and the expression of *OsCNGC16* is reduced [91]. In Arabidopsis, *AtGLR3.4* encodes a glutamate receptor-like protein that enhances cold tolerance by increasing cytosolic Ca^2+^ levels in response to glutamate and cold. Other than Ca^2+^, other signaling molecules, like ROS and NO, are also secondary messengers in plant responses to CS. NO is important as a signal molecule in delivering both plant developmental and environmental signals [92]. In Arabidopsis, the production of NO via nitrate reductase is closely linked to increased freezing tolerance. NO-induced S-nitrosylation of proteins involved in primary metabolism and photosynthesis plays a critical role in protecting against freezing stress by modulating the plant’s antioxidant defense system. Specifically, S-nitrosylation of superoxide dismutase (SOD) boosts its ability to catalyze the conversion of superoxide radicals. Moreover, NO contributes to ROS detoxification through antioxidant pathways [85].

Studies in *Cucurbita pepo* have shown that a mechanism of S-nitrosylation that involves NO plays an important part in ensuring cellular homeostasis by inducing the ascorbate–glutathione (AsA-GSH) cycle under CS [93]. In addition, reduced NO levels, conversely, suppress the expression of cold-induced genes such as C-repeat binding factors (CBFs). Plants produce various ROS when exposed to CS, including O_2_, OH^−^, and H_2_O_2_. Such ROS may damage plant membranes and proteins [94]. Plants have acquired a mechanism of defense against the negative impact of ROS, employing enzymatic antioxidants (glutathione peroxidase (GPX), catalase (CAT), and superoxide dismutase (SOD)) and non-enzymatic antioxidants (glutathione and ascorbic acid). Under stress, SOD catalyzes the dismutation of superoxide (O_2_^−^) into hydrogen peroxide (H_2_O_2_) and oxygen (O_2_). Consequently, H_2_O_2_ is broken down to water and oxygen molecules by CAT, GPX, and ascorbate peroxidase (APX). It has been demonstrated that oxidative stress also impacts RNA and DNA repair systems, which are critical for the adaptation of perennial plants to the CS.

Cold perception is further modulated by photoperiod, light intensity, and circadian rhythms. CBFs are expressed under the control of the circadian clock during cold acclimation. Atmospheric temperature and light intensity changes, and temperature signals, can be monitored by phytotransducers, phytochrome-interacting factors (PIFs). PIF4 in tomatoes perceives light cues and increases the tolerance to low temperatures by upregulating CBF1 [95]. Phytochrome B (Phy B) also contributes to stress adaptation by sensing changes in light and temperature. Studies have shown that CBFs and other cold-activated genes have a rhythmic change in their expression based on daily changes in temperature. This would imply that plants have a system to sense and react to different temperatures. There are the components of circadian rhythm, namely LHY and CCA1, which interact with the promoters of CBF genes and regulate their expression. Mutational analyses of Arabidopsis LHY and CCA1 revealed basal expression of CBF1–3, which revealed the role of CCA1 and LHY in regulating CS [96].

## 4. Phytohormones and Signaling Metabolites in Cold Stress Response

Phytohormones play a crucial role in mediating plant responses to CS, influencing cellular metabolism, membrane stability, and antioxidant defense. Among the key classical hormones involved are abscisic acid, auxin, ethylene, salicylic acid, jasmonic acid, brassinosteroids, strigolactones, and gibberellic acid, and signaling metabolites involved are melatonin and gamma-aminobutyric acid. They have been shown to enhance stress tolerance by regulating gene expression and metabolic pathways. Interactions between these hormones, along with their crosstalk with other signaling pathways, determine the extent of the plant’s ability to adapt and survive under chilling and freezing conditions.

Phytohormone-mediated CS responses involve both endogenous regulatory mechanisms triggered by low temperature and exogenous hormone applications, which have been widely used to enhance stress tolerance under experimental and field conditions. It is important to distinguish between these two processes. Even though they are interconnected, they are mechanistically distinct for regulatory layers, transport, and signaling pathways, resulting in coordinated activation of stress response. Under natural cold conditions, plants dynamically modulate hormone biosynthesis through the ICE–CBF cascade, ROS signaling, and calcium-dependent pathways [97,98]. These endogenous changes reflect intrinsic adaptive mechanisms that fine-tune growth–defense balance [98]. In contrast, exogenous application of phytohormones acts as a priming strategy that artificially enhances or accelerates these signaling pathways, often leading to amplified antioxidant responses, improved photosynthetic stability, and enhanced expression of cold-responsive genes. While exogenous treatments frequently mimic or reinforce endogenous signaling, their effects may differ in magnitude, timing, and regulatory feedback [99]. Therefore, clearly distinguishing these two modes of hormone action is essential for accurately interpreting experimental outcomes and translating laboratory findings into practical agricultural applications.

### 4.1. Abscisic Acid (ABA)

**Figure 3:**
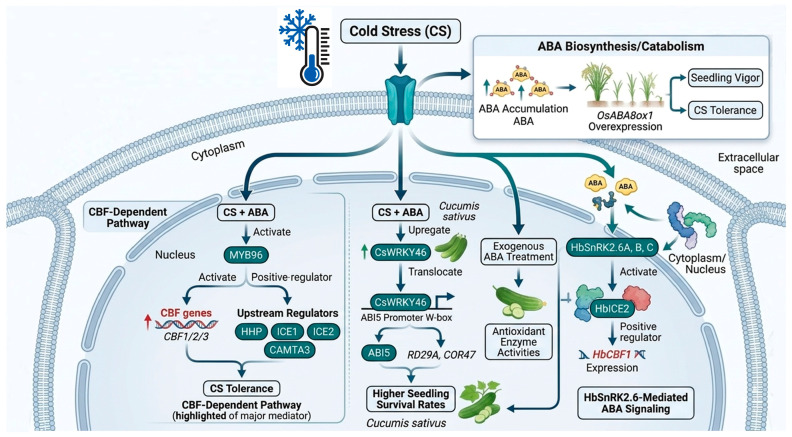
Integrated ABA signaling and cold stress response in plants. The figure highlights how exogenous and endogenous ABA levels influence genetic pathways to enhance cold tolerance. The cold stress (CS) process begins with the sensing of cold stress at the plasma membrane, triggering a cascade of intracellular signaling events. CS induces ABA accumulation. Overexpression of *OsABA8ox1* in rice modulates ABA levels, leading to improved seedling vigor and CS tolerance. In the nucleus, the integration of CS + ABA signals activates MYB96, which acts as a positive regulator to activate CBF genes. This pathway is supported by upstream regulators, including HHP, ICE1, ICE2, and CAMTA3, ultimately resulting in enhanced cold tolerance. The CS and ABA jointly upregulate *CsWRKY46,* which translocates to the nucleus and binds to the ABI5 promoter W-box and triggers the expression of ABI5, RD29A, and COR47, leading to higher seedling survival rates in cucumber. Exogenous ABA enhances antioxidant enzyme activities, which mitigates oxidative damage caused by cold stress. The protein kinases HbSnRK2.6A, B, and C are activated by ABA and activate HbICE2, which drives the expression of HbCBF1, reinforcing the cold stress response through the SnRK2-ICE-CBF signaling axis. Figure created with BioRender.com.

Phytohormones are pivotal regulators of CS responses, modulating the expression of stress-inducible genes and thereby contributing substantially to plant cold adaptation. Among these hormones, ABA is central to CS tolerance. CS activates a range of genes involved in ABA biosynthesis, catabolism, and transport. Adequate ABA accumulation is required for seedling vigor and cold resilience. For example, overexpression of ABA catabolic gene *OsABA8ox1* in rice reduces ABA content but enhances seedling vigor and CS tolerance [100]. ABA functions through both CBF-dependent and -independent pathways, with the CBF cascade serving as a major mediator of cold responses [101,102]. CS- and ABA-induced activation of the MYB96 further enhances CS tolerance by regulating downstream CBF genes and upstream regulators, including HHP, ICE1/ICE2, and CAMTA3 [103]. In *Cucumis sativus*, the transcription factor CsWRKY46 has been shown to enhance CS tolerance in transgenic plants. Studies indicate that both ABA and CS upregulate the expression of CsWRKY46. Transgenic plants overexpressing CsWRKY46 exhibit higher seedling survival rates under CS compared to wild-type plants. This increase in cold tolerance is associated with the upregulation of ABA-responsive TF, such as ABI5, and stress-induced genes like RD29A and COR47. Additionally, CsWRKY46 interacts with the W-box motif in the promoter of ABI5, suggesting that CsWRKY46 regulates CS tolerance through ABA-mediated signaling pathways [104] (Figure 3).

Exogenous application of melatonin or ABA has been shown to enhance cold tolerance by scavenging ROS, boosting non-enzymatic antioxidants such as glutathione (GSH), ascorbate (AsA), total glutathione, and ascorbate, and upregulating key antioxidant enzyme activities, including SOD, CAT, APX, and glutathione reductase (GR) [105]. Moreover, exogenous ABA treatment has been demonstrated to improve cold tolerance by increasing antioxidant enzyme activities. Notably, the protein kinases *HbSnRK2.6A*, *HbSnRK2.6B*, and *HbSnRK2.6C* are further activated by ABA under cold conditions. These kinases, localized in both the cytoplasm and nucleus, interact with HbICE2, a key positive regulator in the cold signaling pathway (Figure 3). This interaction promotes the expression of HbCBF1, a crucial gene involved in CS response. Overexpression of *HbSnRK2.6A* or *HbSnRK2.6B* in Arabidopsis has been shown to enhance cold tolerance by upregulating ABA and *COR* genes, thereby linking *HbSnRK2.6*-mediated ABA signaling to the CS response [106]. These findings underscore the critical role of ABA signaling and its associated regulatory networks in the plant’s ability to respond to and tolerate CS.

Under CS, exogenous ABA treatment not only enhances the activity of antioxidant enzymes such as superoxide dismutase (SOD), catalase (CAT), and peroxidase (POD), but also induces profound biochemical and physiological adjustments [107]. ABA promotes the accumulation of osmo-protectants, including proline, soluble sugars, and glycine betaine, which help maintain cellular turgor and stabilize membrane structures [108]. Additionally, ABA triggers the expression of COR genes and late embryogenesis abundant (LEA) proteins, which protect cellular macromolecules from dehydration damage [109]. At the physiological level, ABA reduces electrolyte leakage and malondialdehyde (MDA) content, thereby preserving membrane integrity [110].

### 4.2. Auxin

The role of auxin in CS responses remains insufficiently characterized, particularly with respect to its biosynthesis, transport, and signaling pathways, in adverse conditions. Auxin is a major plant hormone with a complex and dynamic role under stress conditions. Its activity involves complex interactions with other hormones, environmental messages, and physiological activities. Cold stress at 4 °C causes depletion of auxin in the meristem that leads to loss of quiescent center (QC) identity and premature divisions in the stem cells [111]. Evidence that CS inhibits the inflorescence gravitropic response in Arabidopsis may indicate that CS and auxin are associated [112]. It has been reported that IAA, a major auxin, can increase cold tolerance through reducing ROS accumulation, increasing photosynthetic enzyme activity, and increasing cold-responsive gene expression [72].

**Figure 4:**
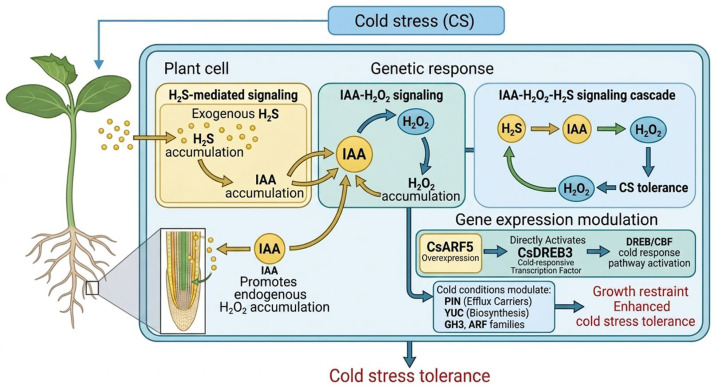
The multi-layered auxin-regulated CS tolerance in plants. This schematic diagram illustrates the proposed signaling network governing CS adaptation in cucumber, highlighting the interplay between the auxin, hydrogen sulfide (H_2_S), and hydrogen peroxide (H_2_O_2_). The signaling occurs within the broader context of the plant’s genetic response to CS, which ultimately leads to growth restraint and enhanced adaptation. Exogenous application of H_2_S enters the plant cell, promoting endogenous H_2_S accumulation and triggering accumulation of endogenous IAA. Conversely, endogenous IAA promotes endogenous H_2_O_2_ accumulation. This interaction creates a positive feedback loop between IAA, H_2_S, and H_2_O_2_ accumulation. The top-right panel simplifies this interaction, showing a proposed pathway where H_2_S triggers IAA, which then stimulates H_2_O_2_ production. Figure created with BioRender.com.

Recent studies indicate that auxin acts as a downstream signaling molecule and interacts with reactive signaling molecules such as hydrogen peroxide (H_2_O_2_) and hydrogen sulfide (H_2_S) during chilling stress [72]. In cucumber seedlings, IAA promotes endogenous H_2_O_2_ accumulation in developing roots and participates in the H_2_S-mediated responses to CS [113]. Zhang et al. [114] found that H_2_O_2_ is a downstream signal of IAA that mediates H_2_S-induced chilling tolerance in cucumber (Figure 4). Furthermore, exogenous H_2_S enhanced IAA and H_2_O_2_ accumulation and induced chilling tolerance, supporting an IAA–H_2_O_2_–H_2_S signaling cascade in low-temperature adaptation [115]. Exogenously applied H_2_S accelerates auxin signaling, resulting in chilling stress tolerance [72]. Several experiments have been conducted on Arabidopsis roots to dissect the molecular and cellular responses of auxin to CS [116]. A recent study showed that chilling stress promotes auxin accumulation and polar transport changes, with auxin gradients and PIN transporter regulation affecting root meristem stability under cold stress, indicating mechanisms by which auxin redistribution contributes to chilling adaptation [117].

Additionally, expression analysis using the auxin-responsive marker IAA2-GUS, along with direct assays for auxin transport, demonstrated that CS primarily influences intracellular auxin transport mechanisms [116]. Shibasaki et al. [118] argue that, unlike auxin signaling, auxin transport is affected by CS. In a study, CS also reduced meristem size and cell division capacity. This could have occurred due to CS reducing the expression of auxin biosynthesis genes and PIN1/3/7 [119]. Intrinsically high levels of auxin have been associated with impaired root system growth and development and reduced nutrient and water uptake under CS [120]. The effects of auxin remain poorly defined in the context of CS, necessitating further studies to elucidate the complex regulatory roles of auxin in CS. Genetic evidence further supports the role of auxin signaling in cold tolerance. Overexpression of the auxin response factor *CsARF5* in cucumber enhances cold tolerance by directly activating the cold-responsive transcription factor *CsDREB3*, thereby linking auxin signaling to the canonical DREB/CBF cold response pathway [121]. This module highlights how auxin signaling can converge with transcriptional regulators to modulate cold-responsive gene expression and antioxidant capacity. Cold conditions modulate the expression and localization of PIN auxin efflux carriers and affect auxin biosynthesis and conjugation genes (e.g., *YUC*, *GH3*, *ARF* families), leading to growth restraint and enhanced stress adaptation (Figure 4). These observations suggest that auxin-mediated growth modulation is an integral component of CS tolerance across taxa.

Exogenous auxin treatment under CS influences several biochemical and physiological processes independent of its effects on antioxidant enzymes [122]. Auxin modulates cell wall remodeling by regulating expansins and xyloglucan endotransglucosylase/hydrolase (XTH) enzymes, thereby maintaining cell wall flexibility and preventing cold-induced rigidity. At the biochemical level, IAA induces the accumulation of heat shock proteins (HSPs) and dehydrins, which act as molecular chaperones to prevent protein aggregation [123]. Physiologically, auxin promotes root system architecture remodeling, including increased lateral root formation and root hair elongation, which enhances nutrient and water absorption under cold conditions [124]. Additionally, auxin delays cold-induced senescence and chlorophyll degradation, thereby sustaining photosynthetic activity for a longer duration [125].

### 4.3. Ethylene (ET)

Ethylene is a gaseous hormone that is very crucial in mediating plant responses to CS. Its function in chilling and freezing tolerance is dualistic, exerting both beneficial and detrimental effects. As a signaling molecule, ET assists plants in adjusting to the CS by facilitating adaptation. CS triggers ET production, which initiates downstream signaling cascades that influence physiological processes and gene expression, which are critical in acclimation to the cold. Excess ethylene production destabilizes the homeostasis in the cells, exacerbating cold-induced damage through lipid peroxidation and tissue senescence.

Moreover, several cold-responsive genes can be suppressed by ethylene signaling, which compromises CS tolerance and hinders cold acclimatization processes [73]. As Sun et al. [126] indicated, cold-acclimated zoysia spp. (*Zoysia grass*) had increased amounts of MDA and EL in the cell membrane, as well as a decreased photosynthetic rate and leaf chlorophyll content. The ethylene response factors (ERFs) play a major role in mediating plant response to different biotic and abiotic stresses. For example, overexpression of *MbERF11* from Siberian crabapple (*Malus baccata*) in Arabidopsis improved salt and cold tolerance by enhancing antioxidant enzyme activity (POD, CAT, SOD), Chl, and proline content and reducing MDA content, whereas overexpression of *MbERF12* also increased salt and cold tolerance, primarily through ROS scavenging mediated by ethylene signaling [127]. Ethylene’s role in CS adaptation is therefore highly complex, involving interactions with other hormone pathways and intricate signaling networks.

ET production increases under low temperatures, leading to a signaling cascade between downstream TFs and activators of positive regulators of CS responses. However, excessive ET signaling can be alleviated by negative feedback mechanisms to establish a balance among cellular components and plant survival during CS. The response of plants to CS is determined by the intricate interplay between positive and negative regulation of the ET signaling pathway. This explains the complexity of the role of ET in CS adaptation [128]. In apple, low temperatures induce ET biosynthesis through the upregulation of *MdACS1* and *MdACO1*. ET signaling is activated through the transcription factor *MdERF1B*, which interacts with *MdCIbHLH1* to form a transcriptional activation complex that enhances *MdCBF1* expression and CS tolerance [129]. ET also modulates postharvest cold tolerance in tomato by regulating *LeCBF1* expression [130].

Exogenous ET or its precursor treatment under CS exerts complex but beneficial effects beyond antioxidant enzyme modulation [131]. At low concentrations, ET promotes the accumulation of compatible solutes such as proline and soluble sugars, enhancing osmotic adjustment. At the biochemical level, ET induces the expression of cold-regulated transcription factors (e.g., CBF/DREB1) and their downstream target genes, which are central to cold acclimation [132]. Physiologically, ET treatment improves seed germination and seedling emergence under chilling conditions by promoting endosperm weakening and radicle elongation [133].

### 4.4. Salicylic Acid

Salicylic acid (SA) plays a key role in plant response to CS by activating defense mechanisms, including the regulation of antioxidant enzymes to mitigate oxidative damage. It also helps in the modulation of gene expression related to stress tolerance, promoting cellular stability and enhancing cold resistance. The biosynthesis of salicylic acid is a product of isochorismate and phenylalanine ammonialyase. Although primarily associated with defense against biotic stress, SA also plays a significant role in CS responses. Supplementation with SA has been shown to mitigate low/chilling temperature damage on various plant species [134]. SA enhanced antioxidant APX, SOD, GPOX, GSH, and GR activities and enhanced chlorophyll fluorescence of *Z. mays* at low-temperature (2 °C) stress [135]. An increase in endogenous SA concentrations induced by CS often results in the expression of cold-responsive genes that mediate stress signaling and cold tolerance. Research conducted by Rivas-San Vicente and Plasencia [76] revealed that SA has been found to enhance photosynthetic efficiency and membrane stability even in the presence of CS, thereby enhancing cold tolerance. However, excessive SA accumulation can inhibit cold acclimation, disrupt signaling, and impair cellular homeostasis. Exogenous acetylsalicylic acid (ASA) application in *Phaseolus vulgaris* prior to CS significantly mitigated cold injury through the upregulation of *CBF3* and *COR47* and increased growth and pigment content [136]. In tomato, SA enhanced cold and oxidative stress tolerance by modulating GA metabolism, CBF1 expression, and antioxidant enzyme activity [137].

SA signaling components interact with negative regulators of CS responses, including ICE1 (inducer of CBF expression 1), a key transcriptional regulator of cold-responsive genes. ICE1 activity is modulated by SA pathways, influencing the plant’s ability to withstand CS [138]. It is important to understand how physiological and biochemical responses mediated by SA benefit plants against CS because this will have a greater impact on plant growth and development. Particularly, the underlying molecular mechanisms through which CS is alleviated by external SA uses are inadequately studied, warranting further research into its regulatory functions.

Exogenous SA treatment under CS elicits a range of biochemical and physiological effects beyond the activation of antioxidant enzymes [139]. SA induces the accumulation of pathogenesis-related (PR) proteins and other defense-related metabolites, which also contribute to cold tolerance through crosstalk between biotic and abiotic stress pathways [140]. At the biochemical level, SA increases the levels of free polyamines (putrescine, spermidine, spermine) and gamma-aminobutyric acid (GABA), both of which act as signaling molecules and osmotic regulators. Physiologically, SA treatment maintains higher net photosynthetic rates by protecting chlorophyll biosynthesis enzymes and reducing photoinhibition [141]. SA also modulates the alternative oxidase (AOX) pathway in mitochondria, reducing ROS production during CS and improving respiratory efficiency [142].

### 4.5. Cytokinins (CKs)

Cytokinins are adenine derivatives with isoprenoid or aromatic side chains at the N6 site, including zeatin (Z), dihydro zeatin (DZ), and N6-(-isopentenyl) adenine (iP) [143]. CK exhibits diverse responses to CS, with both positive and negative effects, and is applied to induce plant responses to CS that promote growth and development, thereby improving their cold tolerance. Endogenous CK levels often decline under CS, contributing to the inhibition of plant growth and development. Exogenous CK administration can offset the adverse effect of CS by promoting cell division, elongation, and differentiation, and enhance plant growth and survival in cold environments [144]. When CK is excessive, it may lead to abnormal growth and susceptibility to cold damage due to the disturbance of the hormonal balance and the impairment of stress adaptation mechanisms. Plant cold tolerance can also be impaired by CKs due to inhibition in the expression of cold-responsive genes or disruption of other hormonal responses to CS [144]. Cytokinin response factor 4 (CRF4) was upregulated by CS treatment (4 °C). In Arabidopsis, CRF4 overexpression lines exhibited increased sensitivity to CS compared with mutants, indicating that CRF4 induction under cold conditions is essential for short-term freezing tolerance [145].

Exogenous CK application under CS alleviates growth suppression through mechanisms extending beyond antioxidant enzyme regulation [146]. CK delays cold-induced leaf senescence by suppressing the expression of senescence-associated genes (SAGs) and maintaining chloroplast ultrastructure. At the biochemical level, CKs enhance the accumulation of chlorophyll and carotenoids, thereby protecting the photosynthetic apparatus from photoinhibition. CKs also upregulate the expression of genes involved in nitrogen assimilation, such as nitrate reductase and glutamine synthetase, improving nitrogen use efficiency under low temperatures [147]. Physiologically, CK treatment maintains stomatal conductance and transpiration rates, preventing excessive reduction in CO_2_ fixation [148]. Furthermore, CKs promote sink strength by enhancing sugar transport from source to sink tissues, supporting the growth of meristems and young leaves.

### 4.6. Jasmonic Acid

Jasmonic acid (JA) and its derivatives, including jasmonate, jasmonate–isoleucine conjugate (JA-Ile), and methyl jasmonate (MeJA), are fatty acid-derived phytohormones synthesized from α-linolenic acid [149]. JA is widely recognized as a multifunctional plant growth regulator, and accumulating evidence underscores its pivotal role in modulating plant responses to CS [39]. Notably, JA levels are higher in senescent leaves compared with non-senescent tissue, suggesting their involvement in senescence-associated stress adaptation. Exogenous JA has been shown to promote leaf senescence and induce senescence-related gene expression, which in turn enhances freezing tolerance in Arabidopsis [150]. In addition, JA alleviates CS by activating ROS-detoxifying enzymes [151] and improving CS tolerance across various plant species.

During CS, the biosynthesis of endogenous JA is activated, which in turn triggers the CBF transcriptional cascade and upregulates downstream cold-responsive genes [152]. The application of exogenous MeJA in wheat has been shown to reduce CS damage by modulating the CBF/DREB1 pathway and promoting the accumulation of COR and dehydrin proteins [152]. In rubber trees, MeJA treatment improves cold tolerance by regulating ICE-like transcription factors, such as HbICE2, and enhancing the expression of key genes in the CBF pathway, including *HbCBF1*, *HbCOR47*, and *HbCBF2* [153].

**Figure 5:**
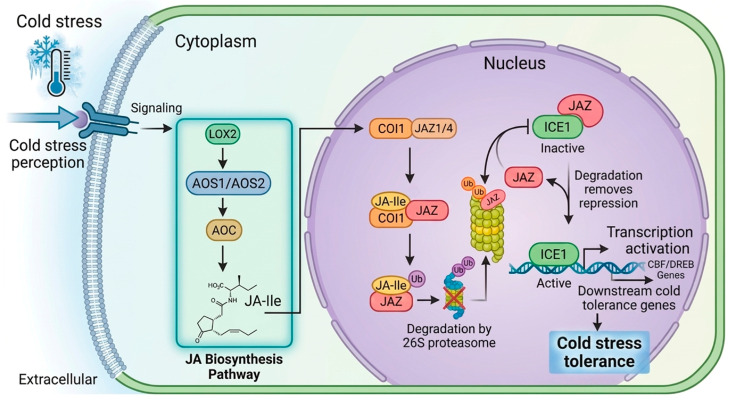
The jasmonate (JA)-mediated cold stress-signaling pathway in plants. Upon sensing cold stress, plant tissues initiate signaling. This leads to the induction or activity of key enzymes involved in JA biosynthesis, including AOS1, AOS2, AOC, and LOX2, resulting in the production of the bioactive jasmonate conjugate JA-Ile. JA-Ile interacts with the COI1 co-receptor and the JAZ repressor proteins (such as JAZI/4). The formation of the JA-Ile/COI1/JAZ complex leads to the ubiquitination and subsequent degradation of JAZ proteins by the 26S proteasome. The degradation of the JAZ repressor prevents its interaction with ICE1, leading to the activation of ICE1, which modulates the expression of downstream genes crucial for conferring cold tolerance. AOS, allene oxide synthase; AOC, allene oxide cyclase; 12-OPDA, 12-oxophytodienoic acid; JA, jasmonic acid; bHLH148, basic helix–loop–helix 148; JAZ, jasmonate zim domain protein; DREB, dehydration-responsive element binding; LOX6, 13-lipoxygenase 6; JA-Ile, jasmonic acid isoleucine; CBF, C repeat binding factor; ICE, inducer of CBF expression; COI1, coronatine insensitive 1. Figure created with BioRender.com.

A central mechanism through which JA enhances cold tolerance is the regulation of the C-repeat binding factor (CBF) pathway. JA positively modulates this pathway by elevating downstream cold-responsive gene expression [150]. The ICE-CBF transcriptional cascade constitutes a core signaling module in plant CS responses [154]. In Arabidopsis, the bHLH transcription factors ICE1 and ICE2 bind to the CANNTG motif in CBF promoters, thereby activating CBF gene expression under cold conditions. At ambient temperatures, however, interactions between JAZ repressors (e.g., JAZ1 and JAZ4) and ICE1/ICE2 suppress the ICE–CBF pathway (Figure 5).

Low-temperature exposure induces the expression of JA biosynthesis genes such as AOS1, AOC, DAD1, and LOX2, leading to increased production of bioactive JA-Ile [154]. JA-Ile is perceived by the JA receptor COI1, which associates with JAZ proteins and triggers their ubiquitin-dependent degradation via the 26S proteasome. The removal of JAZ-mediated repression releases ICE1 and ICE2, ultimately activating the ICE–CBF transcriptional cascade and upregulating cold-regulated genes. This coordinated signaling enhances the plant’s capacity to tolerate CS.

Exogenous JA application influences several biochemical and physiological processes independent of antioxidant enzyme activity [155]. JAs induce the accumulation of secondary metabolites, including anthocyanins, flavonoids, and phenylpropanoids, which function as ROS scavengers and light screens [156]. At the biochemical level, JAs upregulate the expression of dehydrins and cold shock proteins (CSPs), which stabilize nucleic acids and membranes [156]. Physiologically, JA treatment reduces cold-induced electrolyte leakage and lipid peroxidation, preserving membrane fluidity, and also promotes remobilization of storage proteins in seeds and vegetative tissues, providing energy and building blocks for cold acclimation. Furthermore, JA signaling interacts with the circadian clock to optimize cold tolerance responses during the diurnal cycle.

### 4.7. Brassinosteroids

Brassinosteroids (BRs) are a group of polyhydroxylated steroidal phytohormones, including 24-epibrassinolide (EBR) and brassinazole (a BR biosynthesis inhibitor), that have attracted considerable attention for their role in enhancing plant defenses against CS. Acting as essential chemical messengers, BRs modulate cellular activities and integrate diverse signal transduction pathways, thereby coordinating external and internal stress responses [157]. At the biochemical level, BRs promote the synthesis and accumulation of key proteins under chilling conditions, including the β-subunit of ATP synthase [158]. These effects are mediated through BR-dependent regulation of genes involved in biosynthesis and catabolism.

In Arabidopsis, more than 2500 genes are differentially regulated under CS. Importantly, BR biosynthetic genes, such as CPD, DWF4, and BR6ox2, are promptly downregulated following cold exposure [48]. Among these, BR6ox2 exhibits particularly strong suppression, with transcript levels reduced by an order of magnitude at 4 °C, a striking contrast to the typical two- to four-fold changes observed in BR-mediated transcriptional responses [159]. For instance, overexpression of BR biosynthetic genes, including *EnCYP72A14*, *EnCYP90B1*, and *EnDWARF*, has been shown to increase BR levels during CS [160]. When exogenously applied, 24-epibrassinolide (EBR) in *Elymus nutans* helps alleviate CS-induced oxidative damage by reducing ROS and MDA levels. Furthermore, EBR treatment boosts the expression of several cold-responsive genes, such as *EnCOR410*, *EnCBF12*, *EnCS120*, *EnMYB4*, *EnDHN5*, and *EnSDR1* [161]. A similar effect has been observed in *Medicago truncatula*, where BR treatment upregulated cold-responsive genes, including CBF and COR genes such as *MtACS2*, *MtACS7*, and *MtACO1*, contributing to improved low-temperature stress tolerance [162].

**Figure 6:**
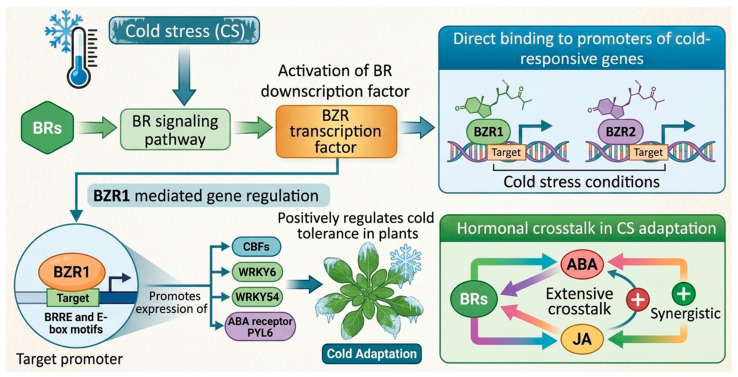
Brassinosteroid-mediated signaling pathways in plant CS adaptation. CS acts as a primary environmental stimulus that modulates the BR signaling pathway. Upon activation, the BR signaling cascade triggers the accumulation and activation of BZR transcription factors (specifically BZR1 and BZR2/BES1), transitioning them into their active dephosphorylated states. Active BZR1 and BZR2 directly bind to the promoters of cold-responsive genes. BZR1 specifically targets BRRE (BR-response element) and E-box motifs within the promoters of downstream effectors. BZR1 promotes the expression of critical regulators, including CBFs, WRKY6, and WRKY54, and PYL6. The BRs, ABA, and JA act synergistically to fine-tune the plant’s physiological response, ultimately leading to enhanced cold tolerance. BRs, brassinosteroids; BZR1/2, brassinazole-resistant ½; CBFs, C-repeat binding factors; ABA, abscisic acid; JA, jasmonic acid; PYL6, pyrabactin resistance 1-like 6; BRRE, BR-response element. Figure created with BioRender.com.

At the molecular signaling level, BRs enhance chilling tolerance by activating downstream mechanisms of the BR signaling pathway, including the BRASSINAZOLE RESISTANT (BZR) transcription factors. BZR1 and BZR2 directly bind to promoters of cold-responsive genes, thereby modulating their expression under CS conditions [163]. Moreover, BR-mediated stress responses are fine-tuned through extensive crosstalk with other phytohormones, particularly ABA and JA, which synergistically contribute to CS adaptation [164]. *BZR1* binds to the BRRE and E-box motifs in the target promoter; promotes the expression of *CBFs*, *WRKY6*, *WRKY54*, and ABA receptor *PYL6*; and positively regulates the cold tolerance in plants [165] (Figure 6). This intricate hormonal interplay underscores the complexity of plant stress-response networks and highlights the central regulatory role of BRs in orchestrating plant tolerance to CS.

Recent evidence further highlights the importance of BRs in CS tolerance and hormonal crosstalk, which has been intensively reviewed recently [166]. Recent studies showed that BR signaling enhances cold tolerance by regulating ROS-producing enzymes and stabilizing photosynthetic performance under chilling stress, while also interacting with ABA-dependent pathways [161,167]. Also, it has been demonstrated that BR-responsive transcription factors directly regulate cold-inducible genes, including components of the ICE–CBF module, thereby integrating BR signaling into the core cold acclimation network [98,161]. These studies reinforce the concept that BRs act not only as growth-promoting hormones but also as key modulators of cold-responsive transcription and redox signaling, often functioning in coordination with melatonin and ABA pathways [98].

Exogenous BR treatment under CS induces multiple biochemical and physiological responses in addition to its well-known effects on antioxidant enzymes [168]. BRs promote the accumulation of osmoprotective compounds, including proline, trehalose, and polyamines, which stabilize membrane lipids and prevent phase transition [169]. At the biochemical level, BRs upregulate the expression of genes encoding RNA chaperones and translational regulators, which maintain protein synthesis under cold stress [170]. Physiologically, BR treatment improves photosynthetic performance by protecting photosystem II (PSII) from photodamage and enhancing carbon assimilation rates [171]. BRs also regulate stomatal aperture and leaf water status, reducing cold-induced wilting [172]. Additionally, BRs promote vascular differentiation and xylem development, facilitating long-distance transport of water and nutrients under cold stress conditions.

### 4.8. Gibberellic Acids

Gibberellic acids (GAs) are a group of tetracyclic diterpenoid carboxylic acids, with GA1 and GA4 serving as key growth hormones in plants. Cold stress inhibits or delays the activity of hydrolytic enzymes during seed germination, processes in which GAs play a critical role [173]. Exogenous GA3 application has been reported to promote seedling development in chickpeas under low-temperature stress, with seed priming enhancing crop establishment, maintaining relative water content, and reducing electrolyte leakage. In addition, studies on two types of rapeseed cultivars revealed that CS increased GA_3_ and ABA levels in one cultivar but not in another, indicating genotype-specific differences in freezing tolerance [174]. GAs, at the molecular level, regulate the expression of CRT/DRE binding factor genes linked to CS tolerance, and contribute to balancing SA and JA signaling in the CBF-mediated CS response [175]. Transcriptomic analyses in Arabidopsis demonstrated significant changes in GA-related genes during CS conditions [36], with the growth regulatory factor (GRF) TF family being identified as a major regulator of GA-dependent growth under CS conditions [36].

**Figure 7:**
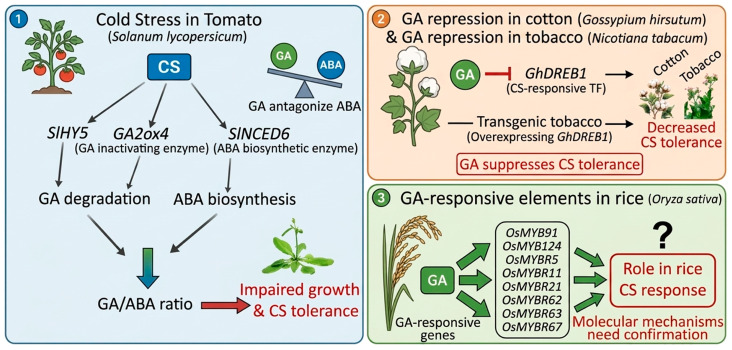
Regulatory crosstalk between gibberellic acid (GA) and ABA in plant response to cold stress. (**1**) Cold stress in tomato: Cold stress induces the expression of SlHY5, GA2ox4 (GA-inactivating enzyme), and SlNCED6 (ABA biosynthetic enzyme). This transcriptional response promotes GA degradation and ABA biosynthesis, resulting in a reduced GA/ABA ratio that negatively impacts growth and CS tolerance. (**2**) GA repression in cotton and tobacco: GA acts as a repressor of GhDREB1, a key cold-responsive transcription factor. High GA levels lead to decreased chilling tolerance in cotton and in transgenic tobacco lines overexpressing GhDREB1. (**3**) GA-responsive elements in rice: Multiple MYB and MYBR transcription factors (OsMYB91, OsMYB124, OsMYBR5, OsMYBR11, OsMYBR21, OsMYBR62, OsMYBR63, and OsMYBR67) are identified as GA-responsive components involved in the rice cold stress response. The precise molecular mechanisms governing these rice-specific elements remain to be fully confirmed. Arrows indicate promotion or induction, and T-bars indicate inhibition or repression. The “?” indicates, functional role is still unknown. Figure created with BioRender.com.

Also, research on the CBF pathway revealed that the cold tolerance mechanisms involve GA-responsive genes [176]. Future studies should be conducted to determine how the GA-related pathways and genes can be used to alleviate CS in plants. GAs generally act antagonistically to ABA and suppress CS tolerance. In tomato, CS induces the expression of *SlHY5, GA2ox4* (a GA-inactivating enzyme), and *SlNCED6* (an ABA biosynthetic enzyme), ultimately reducing the GA/ABA ratio and impairing growth and CS tolerance [177] (Figure 7). GA also represses *GhDREB1*, a CS-responsive transcription factor, thereby decreasing chilling tolerance in cotton and in transgenic tobacco overexpressing *GhDREB1* [178]. A recent study revealed that *OsMYB91*, *OsMYB124*, *OsMYBR5*, *OsMYBR11*, *OsMYBR21*, *OsMYBR62*, *OsMYBR63*, and *OsMYBR67* are GA-responsive elements that play an important role in rice’s response to cold stress. However, their molecular mechanisms still need to be confirmed [179] (Figure 7). Overall, the interaction of GAs seems to be essential for plant development and metabolic regulation under CS conditions.

Exogenous GA application under CS exerts multiple beneficial effects beyond modulating antioxidant enzymes. GA promotes cell elongation and division, thereby mitigating growth inhibition caused by low temperatures [172]. They also enhance the accumulation of soluble proteins and carbohydrates, which function as cryoprotectants [180]. At the physiological level, GA treatment improves photosynthetic efficiency by protecting chlorophyll pigments and maintaining the structural integrity of chloroplasts. Furthermore, GA signaling interacts with DELLA proteins, which are growth repressors that accumulate under CS; exogenous GA degrades DELLA proteins, thus releasing growth arrest [181]. GA also promotes the remobilization of stored reserves in seeds and vegetative tissues, supporting early seedling establishment under chilling conditions [182].

### 4.9. Strigolactones

Strigolactones (SLs) are carotenoid-derived apocarotenoid phytohormones synthesized by cytosolic and plastidic enzymes. They enhance tolerance to stress through regulating physiological, metabolic, and molecular activities. Research has evaluated the impact of GR24 on the growth and development of *B. rapa* plants under CS at a temperature of 4 °C [183]. They discovered that the CS suppressed *B. rapa* seedling development by modulating various physiological and biochemical traits. GR24, when exogenously applied, alleviated the adverse effects of CS by increasing proline accumulation, enhancing cell survival, elevating soluble protein levels, boosting antioxidant activities, and improving photosynthetic performance, while simultaneously reducing ROS generation. According to Raza et al. [120], SLs play a crucial role in increasing CS resistance in Arabidopsis and pea. When comparing wild-type plants with pea *rms4* mutants and Arabidopsis *max* mutants (*max2-1* and *max4-1*), CS in darkness had no impact on photosynthesis in the wild-type plants. However, it severely suppressed photosynthesis in the mutants. These results suggest that SLs shield photosynthesis against the deleterious impact of cold temperature in the dark and promote shoot biomass accumulation [120]. In apple, the *MdD14* gene encodes an SL receptor localized in the cytoplasm and nucleus. Overexpression of *MdD14* enhanced survival under CS, suggesting its role in SL signal transduction and resistance to multiple abiotic stresses, such as salt, drought, and CS [184].

In rice, transcript levels of D17 and D27 remained stable under CS in non-mycorrhizal roots, whereas *Os01g0701500* and *D10* were downregulated, highlighting their involvement in SL biosynthesis and stress adaptation [185]. Current studies are trying to explain how the development of plants is promoted using the high-throughput technology with SL in the presence of CS. The application of the synthetic SL GR24 has been shown to improve freezing tolerance in *Brassica rapa*. This enhancement is associated with the upregulation of genes involved in antioxidant defense, *SOD*, *CAT*, *APX*, and *POD*. Additionally, genes related to NADPH oxidases (e.g., *RbohA–D*, *RbohF–G*), components of the MAPK signaling pathway (MAPK3, MAPK6), and cold-responsive genes like *COR* and *ICE1* are also activated, likely through H_2_O_2_-mediated signaling [72].

In addition to this direct regulation of COR transcription, accumulating evidence highlights crosstalk between SLs and ABA during CS. Disruption of SL signaling alters ABA accumulation and ABA-responsive gene expression, while ABA can feedback-regulate SL biosynthetic genes, suggesting a coordinated regulatory module that fine-tunes stress adaptation. In tomato (*Solanum lycopersicum*), cold exposure induces SL signaling, and application of GR24 enhances cold tolerance by increasing endogenous ABA levels, antioxidant enzyme activities, and expression of ABA- and cold-responsive genes such as CBF1 and HSP70 [186]. Importantly, inhibition of ABA biosynthesis compromises GR24-induced cold tolerance, indicating that SL-mediated cold responses are at least partially ABA dependent [186]. This SL–ABA interaction appears to be conserved across species. SL-deficient mutants in crops such as rice exhibit reduced ABA accumulation and weakened stress-responsive gene expression, suggesting that SLs act upstream of ABA biosynthesis and signaling under abiotic stress conditions [186]. Together, these findings indicate that SLs promote cold tolerance through dual mechanisms: (i) direct activation of cold acclimation pathways via the MAX2–WRKY41–CBF regulatory module, and (ii) indirect enhancement of ABA-dependent stress responses, including antioxidant defense and stress protein accumulation. Coordination between SL and other phytohormones and enhancement of abiotic stress response have been discussed in a recent review article [187]. Strigolactones also enhance cold tolerance via the CBFs module that promotes plant freezing tolerance by releasing WRKY41-mediated inhibition of *CBF*/*DREB1* expression [188].

### 4.10. Melatonin

Melatonin has emerged as a crucial regulator of plant responses to CS. It enhances photosynthetic activity and stomatal conductance while modulating plant hormones, osmotic balance, and metabolic functions under CS conditions [189]. Exogenous melatonin application has been shown to preserve membrane integrity, mitigate CS-induced plasma membrane shrinkage and disruption, and facilitate the recovery of cryopreserved plant tissues. In *Elymus nutans*, melatonin treatment increased ABA levels and enhanced the expression of cold-responsive CBF genes, thereby enhancing cold tolerance [190].

Supporting its protective role, Zhao et al. [191] demonstrated that pretreatment with melatonin helps reduce chilling-induced damage in cucumber seedlings. This effect was linked to the upregulation of genes involved in ABA biosynthesis and the downregulation of genes responsible for ABA catabolism during the early post-treatment phase. Beyond ABA-related pathways, melatonin also influences other stress-responsive genes, such as *CaZat12*, which is implicated in CS adaptation and polyamine metabolism [192]. In pepper plants, melatonin improves root architecture, gas exchange, pigment accumulation, and the expression of CS-related genes, contributing to enhanced overall tolerance [193]. In Arabidopsis, melatonin enhances CS tolerance by inducing the expression of CBF transcriptional activators and their downstream target *COR15a*, as well as other cold-responsive regulators such as *CAMTA1, ZAT10*, and *ZAT12* [194]. Collectively, these findings demonstrate that melatonin enhances plant growth and yield under CS by modulating diverse morpho-physiological and molecular processes. Nonetheless, the precise mechanisms governing melatonin-mediated cold tolerance across different crop species remain to be fully elucidated.

Recent studies have elucidated that the melatonin–BR–H_2_O_2_ tripartite signaling network regulates CS tolerance in many plants, including perennial ryegrass [195]. Melatonin interacts with other phytohormones, particularly auxins, CKs, and BRs, collectively influencing development, growth, and stress tolerance in plants [196]. In tomato and *Arabidopsis*, melatonin application enhances BR biosynthesis and signaling by upregulating BR-related genes (e.g., *DWARF*, *BRI1*), while BR signaling in turn promotes controlled H_2_O_2_ production via NADPH oxidases (RBOHs), which function as a downstream signal rather than a damaging agent [50,197]. Pharmacological inhibition of BR signaling or scavenging of H_2_O_2_ significantly compromises melatonin-induced cold tolerance, indicating that melatonin acts upstream of BR and H_2_O_2_ to orchestrate redox homeostasis and cold-responsive gene expression [50]. Melatonin induces RBOHD-dependent H_2_O_2_ accumulation, which promotes Ca^2+^ signaling and subsequent activation of the CBF cold response pathway, forming a positive regulatory loop that enhances cold tolerance in watermelon [198]. Exogenous melatonin supplement promotes *CmP5CS* expression and positively regulates proline accumulation by activating *CmDREB1A* and *CmDREB1E* in cantaloupe, thereby enhancing CS tolerance [199].

Emerging studies of hormonal stress memory and chromatin dynamics suggest a potential role for melatonin in modulating stress-responsive gene expression via epigenetic mechanisms such as histone modification and DNA methylation in coordination with ROS and hormone signaling networks. However, evidence for melatonin-mediated epigenetic regulation under CS remains limited. Melatonin has been shown to modulate chromatin accessibility by influencing histone acetylation and methylation states at cold-responsive loci, including *CBF* and *COR* genes. In *Arabidopsis*, melatonin treatment enhances H3K4me3 and H3K9ac enrichment at promoters of cold-inducible genes, correlating with sustained transcriptional activation during prolonged or repeated cold exposure [200]. These chromatin modifications persist beyond the initial stress period, suggesting a role for melatonin in cold stress priming and transcriptional memory [98].

### 4.11. Gamma-Aminobutyric Acid

Gamma-aminobutyric acid (GABA) is a non-proteinogenic amino acid that functions as an important signaling metabolite in plants, particularly under abiotic stress conditions. It is synthesized primarily from glutamate by the calcium/calmodulin-regulated enzyme glutamate decarboxylase (GAD) and finally metabolized by GABA transaminase (GABA-T) and succinic semialdehyde dehydrogenase (SSADH), linking cytosolic Ca^2+^ dynamics with metabolic and signaling responses under stress conditions [201]. GABA interacts with phytohormones to regulate plant growth, development, and stress tolerance and plays a crucial regulatory role in plant adaptation to CS by coordinating antioxidant defenses, metabolic balance, energy homeostasis, and cellular integrity [202]. Cold stress has been shown to induce rapid GABA accumulation, suggesting a role in stress perception and downstream signaling.

Increasing evidence indicates that GABA contributes to cold tolerance by modulating cytosolic pH, carbon–nitrogen balance, and ROS homeostasis. GABA has also been implicated in regulating antioxidant enzyme activity and stabilizing cellular membranes under low-temperature stress. Compared with CS, GABA + CS improved the activities of antioxidant enzymes and the contents of antioxidants, thereby increasing wheat antioxidant capacity and decreasing MDA content under CS [47]. Application of GABA mitigated chilling-induced damage in tomato through the protection of chloroplast ultrastructure, altering several metabolism pathways and reducing oxidative damage during the reproductive stage [203]. 

At the molecular level, GABA signaling is closely integrated with Ca^2+^, ROS, and abscisic acid (ABA) pathways. Cold-induced Ca^2+^ influx activates GAD, promoting GABA biosynthesis, while ABA-dependent signaling further enhances this response. GABA has been shown to regulate the activity of aluminum-activated malate transporters (ALMTs), thereby influencing anion fluxes and membrane potential during stress signaling [80]. Key components of GABA metabolism and transport, including GAD, GABA transaminase (GABA-T), and ALMTs, have been implicated in stress tolerance and represent important regulatory nodes linking metabolic and hormonal networks under CS.

Crosstalk between GABA and phytohormonal pathways is emerging as a key regulatory feature of CS responses. GABA accumulation is often coordinated with abscisic acid (ABA) signaling, particularly through shared calcium- and ROS-dependent pathways, and may act synergistically with ABA to enhance stress tolerance during freezing and cold acclimation. Although direct CRISPR/Cas9-based manipulation of GABA-related genes for cold tolerance remains limited, emerging genome editing studies targeting GAD and GABA-T genes highlight their potential as future targets for enhancing low-temperature resilience. Together, these findings support the inclusion of GABA as an important component of the broader hormonal and signaling network regulating plant responses to CS.

## 5. Practical Application and Limitations of the Phytohormones

At the end, after individual hormonal discussion, we highlighted the practical applications and translational perspectives of the phytohormone. Recent advances in phytohormone-mediated cold stress signaling provide clear opportunities for improving crop cold tolerance under field conditions. Exogenous application of phytohormones such as melatonin, SA, GABA, and BRs through seed priming or foliar sprays has consistently enhanced germination, antioxidant capacity, photosynthesis, and yield stability under chilling stress in major crops, including rice, maize, tomato, wheat, and horticultural species [41,46,47,92,161]. These approaches are cost-effective, easily adoptable, and compatible with current agronomic practices. Beyond short-term interventions, hormone-informed breeding and genome editing offer durable solutions. Key regulatory modules, including ICE–CBF, DELLA–GA, BZR1–BR, and SL–ABA signaling pathways, represent promising targets for marker-assisted selection and CRISPR/Cas-based crop improvement aimed at enhancing cold tolerance without severe growth penalties [49,188,204,205]. Emerging evidence also highlights the importance of phytohormone-mediated stress memory and priming, which may provide sustained protection against recurrent cold events under fluctuating climates [98,206]. However, broader adoption requires more multi-location field trials and crop-specific optimization. Integrating molecular insights with field validation will be critical for translating phytohormone-based strategies into climate-resilient agriculture.

However, the limitation is that the effects of ABA on cold tolerance are highly context-dependent, varying with tissue type, stress intensity (chilling vs. freezing), and interaction with other hormone-mediated growth repression. Although auxin generally promotes cold acclimation, several studies report growth inhibition under prolonged chilling, indicating a trade-off between stress tolerance and developmental progression. Reports on ET function remain conflicting, with both positive and negative roles described, likely reflecting species-specific signaling hierarchies and interaction with JA and ABA pathways. These contrasting observations suggest that phytohormone-mediated cold responses are not universally conserved and must be interpreted within specific genetic and environmental contexts.

While phytohormones enhance cold tolerance, their regulatory effects for practical application often involve trade-offs between stress adaptation and growth and productivity. For example, elevated JA-induced cold acclimation frequently suppresses cell division, leaf expansion, and biomass accumulation. ABA and SA can similarly reduce photosynthetic carbon assimilation or accelerate senescence in a dose-dependent manner. Similarly, reduced GA activity promotes stress resilience at the expense of cell expansion and developmental progression. These growth–defense trade-offs necessitate careful optimization of hormone concentration, application timing, and delivery method, as excessive levels may lead to growth inhibition or metabolic imbalance. Emerging strategies such as tissue-specific gene expression, chemical priming, and manipulation of negative regulators offer potential avenues to decouple stress protection from growth inhibition. However, crop-specific and context-dependent data remain limited, highlighting a critical direction for future research.

## 6. Hormonal Crosstalk in Response to Cold Stress

Plant responses to CS involve complex interactions among multiple hormonal pathways, mediated by calcium signaling cascades, TFs, and diverse secondary signaling molecules. Among these, ABA acts as a central regulator, coordinating the integration of several hormonal signals to optimize plant adaptation to cold environments. Understanding the molecular mechanisms underlying hormonal crosstalk during CS is therefore crucial for improving cold tolerance and ultimately enhancing agricultural productivity in low-temperature conditions. A defining feature of CS adaptation is the balance between defense activation and sustained growth. Importantly, defense responses depend not only on the individual contributions of hormones but also on the type of crosstalk—positive or negative—occurring between their respective signaling pathways. Networks involving ABA, GA, JA, and BRs converge and interact at multiple regulatory nodes, including hormone-responsive transcription factors, to fine-tune plant defense responses [207]. Additionally, in the context of fig leaf gourd grafting onto cucumber seedlings, ABA has been found to activate the WRKY41/WRKY46-miR396b-5p-TPR module, which is involved in the CS response [208]. In grafting-induced cold tolerance, ABA not only promotes the accumulation of melatonin and MeJA, but also stimulates ABA accumulation through a positive feedback loop, thereby further enhancing cold tolerance [209].

A well-characterized example of GA–ABA crosstalk in CS adaptation involves the CBF1 regulatory pathway. In Arabidopsis, CBF1 promotes the accumulation of the DELLA protein RGA, reduces cellular GA levels, and activates GA2-oxidases. Consistent with this mechanism, loss-of-function mutations in DELLA proteins GAI and RGA impair freezing tolerance, demonstrating that DELLAs act via the CBF1 pathway to support cold adaptation [210]. ABA–JA interactions also play a pivotal role in coordinating plant development and defense. JAZ–MYC2 components act as central integrators, while ABA receptor PYRABACTIN RESISTANCE1-like proteins (PYLs) modulate JA-mediated metabolic reprogramming in both tobacco and Arabidopsis. This interplay regulates elicitor-induced metabolic and developmental changes, underscoring the importance of ABA–JA crosstalk in stress responses. Cytokinins intersect with auxin and ABA pathways to modulate the expression of cold-responsive genes involved in stress signaling, antioxidant defense, and osmotic regulation. Through these interactions, cytokinins contribute to cellular homeostasis and improved CS adaptation and tolerance [144,211].

Together with ABA, SL contributes to a coordinated hormonal module that integrates ROS signaling, growth restraint, and stress tolerance, underscoring the importance of SL–ABA interactions in optimizing cold stress responses. A recent study in wheat oligo-tillering mutant *ot1* demonstrates coordinated upregulation of SL and ABA synthesis/signaling pathways, further supporting the relevance of SL–ABA interactions in crops [212]. Coupled with mechanistic insights on SL regulation of freezing tolerance via CBF signaling, these findings point to SL–ABA crosstalk as an integral component of hormonal networks underpinning cold stress adaptation [188].

**Figure 8:**
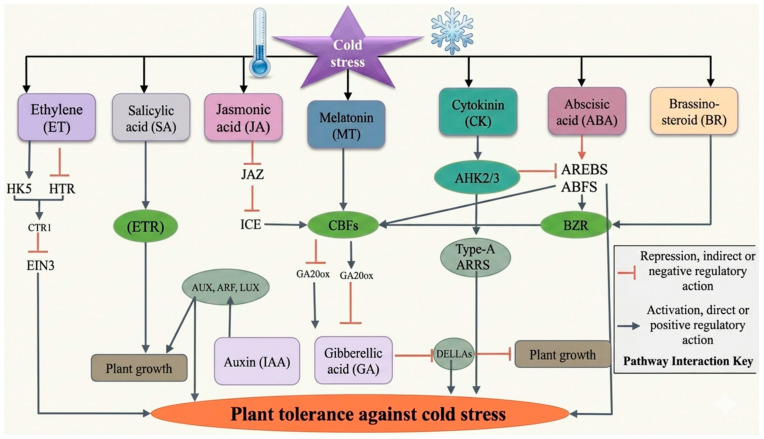
Phytohormone signaling in plants during cold stress. Key components of ABA, JA, BR, IAA, and SA pathways can positively enhance CBF-dependent pathways. Conversely, GA, CK, and ET pathways primarily regulate cold stress responses through CBF-independent mechanisms [213]. ARRs, Arabidopsis response regulators; BZR, brassinazole resistant; CTR1, constitutive triple response 1; CBFs, cold binding factors; ICE, ethylene insensitive; ETR1, ethylene receptor 1; GA20ox, GA20 oxidase; GA2ox, GA2 oxidase; HKs, histidine kinases; ICE, inducer of CBF expression; JAZ, jasmonate zim-domain; AHKs, membrane-located CK receptor; ABFs, ABRE binding factors; AREBs, ABRE binding proteins; AUX, auxin; ARF, auxin response factor. Figure created with BioRender.com.

The growth–defense balance is further modulated by JA–BR interactions. While JA generally suppresses plant growth, BR promotes above-ground development. Low BR concentrations induce anthocyanin accumulation, defense responses, and the expression of *OsDI1* and *OsDWARF* during early and late stages of BR biosynthesis, respectively. Conversely, high BR levels activate BR signaling cascades involving BRI1, BAK1, and BR-responsive TFs such as BES1 and BZR1, which regulate downstream gene expression and abiotic stress responses. Notably, elevated BR concentrations suppress endogenous JA and BR biosynthesis, whereas JA inhibits BR production, indicating a tightly regulated JA–BR feedback network that governs defense–growth trade-offs under stress [48,157]. Collectively, these findings demonstrate that plant responses to CS are mediated by an intricate hormonal network involving ABA, GA, JA, BRs, cytokinins, and auxin. Deciphering these molecular interactions will provide valuable insights for developing strategies to enhance cold tolerance and improve crop performance in chilling-prone environments. Collectively, hormonal crosstalk under cold stress is highly dynamic rather than linear. Conflicting outcomes reported across studies likely arise from differences in experimental systems, stress regimes, and phenotypic endpoints, underscoring the need for standardized comparative frameworks. The major positive and negative regulatory pathways influenced by CS-induced phytohormone signaling are summarized in Figure 8.

## 7. Phytohormone Signaling Networks in Cold Stress

Cold stress tolerance in plants is governed not by isolated hormone actions but by highly interconnected phytohormone signaling networks that integrate environmental perception with growth–defense trade-offs in which multiple hormones, redox signals, and secondary messengers interact in a coordinated manner. Research evidence indicates that cold acclimation and stress memory responses are organized into tripartite and multipartite signaling modules among ABA, auxin, JA, BRs, SLs, ET, SA, CK, and melatonin, together with secondary messengers such as Ca^2+^, ROS, and H_2_S that enable rapid perception of low temperature, signal amplification, and fine-tuned regulation of downstream physiological and transcriptional responses [52,120,213].

Several tripartite signaling networks have emerged as central regulators of cold stress tolerance in plants. The ABA–ROS–Ca^2+^ module represents a core signaling axis in which cold-induced membrane rigidification triggers Ca^2+^ influx, promoting ROS production and ABA accumulation. These signals converge on SnRK2/OST1 kinases and the ICE1–CBF transcriptional cascade, ultimately activating cold-responsive (COR) genes and enhancing osmotic adjustment, antioxidant capacity, and chilling and freezing tolerance in plants [34,106,214]. Similarly, in the melatonin–BR–H_2_O_2_ network, melatonin modulates BR signaling and controls H_2_O_2_ production to balance antioxidant defense, growth restraint, and activation of cold-responsive gene expression [195,196]. In addition, an auxin–H_2_O_2_–H_2_S signaling cascade has been implicated in chilling tolerance, highlighting how classical growth hormones integrate with redox signaling molecules and gasotransmitters to regulate ROS homeostasis and stress adaptation [115].

Beyond these tripartite modules, multipartite phytohormone networks operate through shared regulatory nodes that integrate multiple hormonal inputs. ABA, JA, BRs, GAs, ET, and SL converge on key transcriptional and post-translational regulators, including ICE1/CBFs, DELLA proteins, JAZ repressors, SnRK2 kinases, and ROS-scavenging enzymes [215]. Antagonistic interactions between growth-promoting hormones (e.g., GA and auxin) and stress-adaptive hormones (e.g., ABA and JA) allow plants to prioritize survival under cold stress, often at the expense of growth. ETs and SLs further modulate this balance by influencing ROS signaling, hormone sensitivity, and transcription factor stability. Recent studies demonstrate that SLs enhance cold and freezing tolerance through ABA-dependent mechanisms involving HY5-regulated autophagy, WRKY-mediated control of CBF/DREB1 expression, and stress-responsive protein turnover [45,188].

Importantly, the output of these signaling networks is highly context dependent, varying with temperature severity (chilling versus freezing), tissue type, developmental stage, and species. For example, ABA-dominated networks are particularly important during freezing tolerance and cold acclimation, whereas auxin- and BR-associated modules play prominent roles in chilling tolerance and post-stress recovery. Understanding these integrated phytohormone signaling networks provides a conceptual framework for identifying key regulatory hubs that are suitable targets for genetic manipulation or chemical priming to enhance cold stress tolerance in crops.

## 8. Integrative Synthesis and Decision-Oriented Framework

Although individual phytohormones and signaling metabolites, discussed earlier, contribute distinctly to CS tolerance, increasing evidence indicates that these signals converge on a limited number of shared regulatory hubs. Prominent among these are the ICE–CBF transcriptional module, SnRK2/*OST1* signaling, DELLA growth repressors, JAZ–COI1 jasmonate signaling, and ROS/Ca^2+^ integration nodes [210,214,216]. These shared components act as decision points that coordinate growth restraint, redox balance, stress signaling, and transcriptional reprogramming under low-temperature conditions.

Importantly, the relative contribution of specific hormonal pathways depends on the type of CS and developmental context. For seedling establishment under chilling stress, pathways that maintain membrane integrity, photosynthetic capacity, and cellular homeostasis, such as auxin transport (PIN–ARF modules), ABA–SnRK2 signaling, and CBF/DREB1-*COR* signaling pathway, and melatonin-mediated redox regulation, appear especially effective [217,218]. In contrast, freezing tolerance relies more heavily on transcriptional reprogramming via the ICE–CBF regulon and JA-JAZ and GA-*DELLA* module-mediated growth suppression [38].

Hormonal crosstalk enables fine-tuning of these responses, allowing plants to balance survival with growth. Genetic evidence from both model plants and crops suggests that regulatory nodes such as *OST1*, ICE1, CBFs, DELLA proteins, JAZ repressors, melatonin biosynthetic enzymes (SNAT/ASMT), and GABA metabolic enzymes (GAD) represent promising targets for genome editing or marker-assisted breeding. Table 3 summarizes an integrative overview linking phytohormones, regulatory nodes, phenotypic outcomes, and candidate targets, thereby offering a decision-oriented framework to guide the selection of pathways and candidate targets for improving CS tolerance in plants.

## 9. Genetic Engineering of Phytohormone Pathways for Cold Stress Tolerance

**Figure 9:**
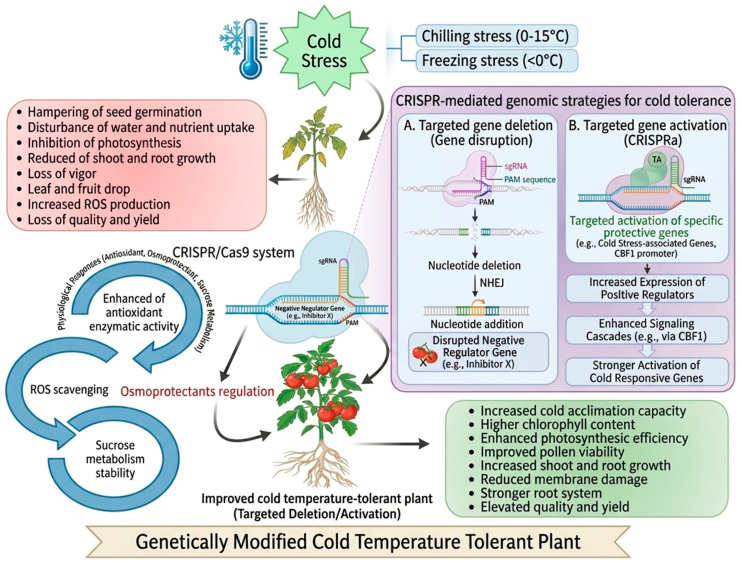
Schematic overview of CRISPR/Cas9-based genome-editing-mediated enhancement of cold stress tolerance in plants. The illustration depicts how low or fluctuating temperatures disrupt normal plant functions. This disruption leads to lower germination rates, impaired flowering or infertility, diminished photosynthetic efficiency, and ultimately, reduced crop yields. CRISPR/Cas9-mediated targeted modifications of stress-responsive genes and regulatory elements alter protein function, thereby reinforcing biochemical and physiological resilience to cold stress (figure created with BioRender.com).

Cutting-edge technologies, such as genetic engineering, CRISPR-based genome editing, transgenic overexpression, and single-cell transcriptomics, have dramatically advanced our understanding of stress signaling in spatial and temporal dimensions [219,220,221]. Figure 9 summarizes the impacts of CS on plant physiological and molecular processes and illustrates how genetic engineering approaches, including CRISPR/Cas9-mediated genome editing and transgenic manipulation, have been applied to dissect and improve CS tolerance. Table 4 provides an overview of plant species in which phytohormone-related genes have been genetically modified to enhance resilience to CS.

### 9.1. CRISPR/Cas9-Based Functional Dissection of Cold-Responsive Regulators

Recent developments in CRISPR/Cas genome editing provide opportunities to manipulate phytohormone-mediated CS responses, where Arabidopsis serves as a model plant for dissecting the molecular and physiological mechanisms underlying CS adaptation through CRISPR/Cas [222]. In the context of cold stress, the C-repeat binding factors (CBFs) represent a key gene family controlling downstream cold-responsive (COR) genes and enhancing freezing tolerance [204,223]. Using the CRISPR/Cas9-method, targeted editing of key regulatory genes like ICE1, CBF1, CBF2, and CBF3 can enhance cold-responsive gene expression and generate single, double, and triple mutant lines of *A. thaliana* [204,222], whereas modification of ABA signaling components (e.g., SnRK2s or PYR/PYL receptors) can improve stress signaling efficiency. Similarly, editing DELLA proteins in the gibberellin pathway, JAZ proteins in the jasmonate pathway, or BZR1 in the brassinosteroid pathway offers opportunities to balance growth and stress tolerance [224,225].

Mutations in CBF-generated *cbf1 cbf2 cbf3* triple mutants exhibited heightened susceptibility to freezing compared with wild-type plants, confirming the collective role of CBF genes in freezing tolerance. It was demonstrated that *cbf2-cbf3* double mutants were more prone to freezing injury than *cbf1-cbf3* mutants, which were tolerant to freezing stress, indicating that the CBF2 gene was more significant in the response to freezing as compared to the other two genes [204,222]. The triple mutants with the *CBF1*, *CBF2*, and *CBF3* genes knocked down with CRISPR were more susceptible to freezing stress when they were exposed to the cold acclimation treatment than the double (*CBF1,3*) and single (*CBF3*) mutants [204]. To study the mechanism of low-temperature tolerance in tomato (*Solanum lycopersicum*), Li et al. [205] used CRISPR/Cas9 technology to generate *slcbf1* mutants, which exhibited decreased tolerance to low temperatures. *SlCBF1* knockout led to a reduction in the concentrations of MeJA, ABA, and zeatin riboside, while markedly enhancing the concentration of IAA [205]. In rice, CRISPR/Cas9-mediated editing of hormone-associated regulators, including *OsPIN5b*, *GS3*, and *OsMYB30*, improved cold resilience and agronomic performance. *OsPIN5b*, an auxin transporter involved in auxin homeostasis, was shown to influence panicle development and cold tolerance, underscoring the importance of auxin distribution in CS adaptation [226].

### 9.2. Transgenic Manipulation of Phytohormone-Related Genes Under Cold Stress

In addition to genome editing, transgenic overexpression studies have provided valuable insights into hormone-mediated cold tolerance. In banana (*Musa acuminata* L.), upregulation of the aquaporin gene *MaSIP21* reduced leaf area and plant height under normal growth conditions. Xu et al. [227] reported that *MaSIP21* positively influenced drought and CS tolerance by enhancing osmotic adjustment, chlorophyll content, and membrane stability, and reducing H_2_O_2_ and MDA accumulation, alongside modulating ABA and GA levels. Also, upregulation of *MaPIP27* improved the CS tolerance by strengthening biosynthetic and responsive gene expression [228].

Auxin-related genes also play important roles in CS tolerance. Overexpression of *GmNAC20* significantly enhanced CS tolerance in rice by modulating abiotic stress-related genes and regulating the expression of auxin-related genes [229]. In *Medicago falcata*, upregulation of *MfAIR12* (auxin-induced in root culture) enhanced tolerance to CS by increasing the accumulation of H_2_O_2_ in the apoplast, thereby regulating ascorbate homeostasis and activating the CBF cold response pathway [230].

ABA- and JA-mediated pathways are further implicated in woody perennials. In apple, overexpression of *MdABI4* enhanced cold tolerance through ABA-dependent interaction with *MdICE1*, promoting *MdCBF1* expression, whereas *MdJAZ1* and *MdJAZ2* negatively regulated this response [231]. On the other hand, the jasmonate ZIM-domain (JAZ) proteins MdJAZ1 and MdJAZ2 appear to inhibit MdABI4’s activity, ultimately reducing CS tolerance in apples [231]. In grapevine, overexpression of *VaPAT1* (phytochrome A signal transduction 1) and *VaIDD3* (indeterminate-domain 3) increased cold tolerance by enhancing *VaLOX3* expression and JA accumulation [232,233]. Conversely, repression of *VaPAT1* using dominant-negative constructs or CRISPR-mediated mutagenesis reduced JA levels and cold tolerance [233].

*A. thaliana* overexpressing the CK biosynthetic gene DEX: IPT showed improved tolerance to CS, which was associated with higher levels of auxin in the apices and greater levels of SA and CK in the shoots [234]. Ethylene-responsive transcription factors (ERFs) regulate both ethylene- and ABA-mediated signaling and interact with GCC box and DRE components in low temperatures, thereby increasing the CS tolerance [235]. Overexpression of *BpERF13*, *MdERF1B*, and *PtrERF9* enhanced chilling and freezing tolerance by regulating ROS homeostasis and activating CBF-dependent pathways across multiple species [17,236]. Similarly, upregulation of *MdERF1B* improved CS tolerance in Arabidopsis and apple seedlings [129]. *PtrERF9* served as a downstream functional component of the ethylene signal transduction pathway and increased the resistance to CS by regulating the activity of *PtrGSTU17* and maintaining the equilibrium of ROS in cells [236].

### 9.3. Limitations, Biosafety, and Regulatory Considerations

Despite its power and precision, CRISPR/Cas9-mediated genome editing carries several technical and biosafety challenges that warrant careful consideration in plant applications. A primary concern is off-target editing, where the Cas9/sgRNA complex introduces unintended mutations at genomic sites with partial sequence similarity, potentially affecting gene function and phenotypes unrelated to the intended trait [237]. Limitations in delivery methods, such as genotype-dependent transformation efficiency and low rates of homology-directed repair, further constrain precise editing applications. Precise editing via homology-directed repair (HDR) is also inefficient in plants due to preferential use of non-homologous end joining pathways, limiting targeted gene replacement.

Beyond technical considerations, regulatory frameworks governing genome-edited crops vary globally, influencing research translation and commercialization. While some jurisdictions exempt DNA-free edited plants from GMO regulations, others impose strict oversight. Addressing these challenges will require advances in guide RNA design, whole-genome off-target profiling, robust phenotyping, and harmonized regulatory policies [238]. Public acceptance and ethical considerations also play a role in shaping investment and policy landscapes, particularly for food and environmental applications. Addressing these challenges will require a combination of improved specificity and detection methods, like gRNA design informed by machine learning, whole-genome off-target profiling, and rigorous phenotyping, alongside harmonized regulatory guidance to support responsible application of CRISPR technologies in crop improvement [239]. Several regulatory nodes summarized in Table 2, including ICE1, *OST1*/SnRK2, DELLA proteins, BZR1, JAZ repressors, and melatonin biosynthetic genes (SNAT/ASMT), represent promising CRISPR/Cas9 targets for improving CS tolerance in plants.

**Table 4:** Genetic and hormonal manipulation of phytohormone pathways conferring cold stress tolerance in plants.

Plant Species	Stress Type	Regulated Genes	Hormone Pathways	Engineering Approach	Key Mechanism	References
*Arabidopsis thaliana*	Chilling/freezing	*DTX/MATE*	ABA	Transgenic overexpression	Enhanced cold tolerance through increased antioxidant enzyme activity, maintenance of leaf water content, reduced ion leakage, and upregulation of ABA-responsive genes (*ABF4*, *RD29B*, *SOS1*, *CBL1*).	[240]
*Arabidopsis thaliana*	Chilling	*SsKAI2*	ABA	Transgenic overexpression	Increased expression of cold acclimation and ABA signaling genes, improving chilling tolerance but conferring hypersensitivity to ABA.	[241]
*Arabidopsis thaliana*	Cold acclimation	*ClWRKY20*	ABA, JA, ethylene, and auxin	Transgenic overexpression	Enhanced cold tolerance via activation of ABA signaling and coordinated induction of JA, ET, and auxin signaling pathways.	[242]
*Arabidopsis thaliana*	Freezing	*SLR1* and *OsGA20ox1*	GA	Transgenic overexpression	Reduced active GA levels promoted freezing tolerance through activation of *OsGA2ox1* and suppression of *OsGRF6*, indicating GA-mediated growth restraint.	[243]
*Oryza sativa*	Chilling	*OsMYB30*, *OsPIN5b* and *GS3*	Auxin	CRISPR/Cas9 knockout	Edited lines exhibited improved cold tolerance and productivity, highlighting the role of auxin transport and transcriptional regulation in cold resilience.	[226]
*Zea mays*	Cold acclimation	*ZmPgb1.1*	BR	Transgenic overexpression	Modulation of BR biosynthesis and signaling genes reduced ROS accumulation and enhanced cold tolerance via BR-dependent MAPK pathways.	[244]
*Solanum lycopersicum*	Chilling	*SlCYP90B3*	BR	Transgenic overexpression	Improved chilling tolerance during cold by regulating *SlCBF1* expression and enhancing antioxidant enzyme activity.	[245]
*Solanum lycopersicum*	Chilling	*SlNPR1*	SA	Loss-of-function mutant	Mutant plants showed increased cold susceptibility, indicating a positive role of SA signaling in chilling tolerance.	[246]
*Solanum lycopersicum*	Chilling	*SICBF1*	Auxin, ABA, and JA	CRISPR/Cas9 knockout	Loss of *SlCBF1* reduced chilling tolerance; increased electrolyte leakage and H_2_O_2_ accumulation; decreased ABA, MeJA, and zeatin riboside levels; and increased IAA.	[205]
*Solanum lycopersicum*	Chilling	*SlF3HL*	JA	Transgenic overexpression/suppression	Overexpression enhanced chilling tolerance through JA signaling, while suppression increased ROS, MDA accumulation, and membrane damage.	[247]
*Malus domestica*	Freezing	*MdABI4*	ABA and JA	Transgenic overexpression	Integrated ABA and JA signaling to activate the JAZ–ABI4–ICE1–CBF regulatory module, enhancing cold tolerance.	[231]
*Malus domestica*	Chilling	*MdBBX37*	JA	Transgenic overexpression	Promoted *MdCBF1* and *MdCBF4* expression through ICE1 activation; JA-dependent cold tolerance regulated via BBX37–ICE1–CBF module.	[248]
*Prunus persica*	Chilling	*PpSOD*, *PpPOD*, and *PpAPX*	ABA	Transgenic overexpression	ABA-induced H_2_O_2_ production triggered nitric oxide signaling and stomatal closure, contributing to enhanced cold tolerance.	[249]
*Cucumis melo*	Chilling	*CmNCED3*, *CmNCED3-2*, *CmADC*, *CmSAMDCs*, *CmSPDS2*	ABA	Exogenous hormone treatment	ABA application enhanced antioxidant enzyme activities and reduced membrane lipid peroxidation, mitigating chilling-induced oxidative damage.	[250]
*Brassica napus*	Freezing	*BnARF16*, *BnSAUR60*, *BnSAUR62*, *and BnCKX3*	Auxin, CK	Transgenic overexpression	Improved cold tolerance associated with induction of stress-responsive TFs (*NAC56*, *NAC29*, *DREB1B*, *ERF70*) and modulation of auxin–cytokinin balance.	[251]
*Solanum lycopersicum*	Chilling	*APX*, *MDAR*, *DHAR*	ABA, JA, SA, and ET	Not available	Improved chilling stress tolerance.	[252]
*Saccharum officinarum*	Chilling	*SoDSP2*	ABA	Transgenic overexpression	Gene expression associated with ABA, GA, auxin, and ET signaling was consistently downregulated. A modest upregulation of BZR1 was observed in the leaves.	[253]

The table integrates genome-edited lines, transgenic approaches, and hormone-based treatments; the engineering approach and dominant cold context (chilling vs. freezing) are explicitly indicated to improve clarity and comparability.

## 10. Conclusions and Future Perspectives

Cold stress, encompassing both chilling (0–15 °C) and freezing (≤0 °C) conditions, is one of the major abiotic constraints limiting plant growth, development, and productivity, particularly in regions exposed to low temperatures. This review synthesizes current evidence demonstrating that phytohormones and signaling metabolites are central regulators of CS tolerance, which is governed by tightly interconnected signaling networks, with shared regulatory hubs such as the ICE-CBF module, SnRK2 kinases, DELLA proteins, JAZ/COI1 complexes, and ROS-Ca^2+^ signaling nodes coordinating adaptive responses, rather than isolated pathways. Importantly, the relative contribution of these pathways differs between chilling-dominant processes (e.g., seedling establishment and photosynthetic stability) and freezing-dominant processes (e.g., cold acclimation and cellular dehydration tolerance).

From a translational perspective, future research and breeding efforts should prioritize stress-context-specific targets. For improving chilling tolerance, particularly at early developmental stages, manipulation of ABA-SnRK2 signaling, *OST1*/SnRK2 activation, auxin-mediated growth regulation, and ROS-scavenging pathways appears most promising, supported by both chemical and genetic evidence in both model plants and crops. Enhancing freezing tolerance will likely require precise modulation of transcriptional regulators involved in cold acclimation, including ICE-CBF regulation, downstream COR gene expression, and GA-DELLA modulation, where strong genetic validation already exists in model plants and is increasingly emerging in crops.

Growth-related hormones such as auxin, GA, and BRs primarily modulate cold tolerance indirectly by balancing growth restraint and cellular homeostasis, highlighting the importance of growth–stress trade-offs rather than absolute stress resistance. Jasmonates, ethylene, and salicylic acid act mainly as modulators of stress amplification and fine-tuning, often through transcriptional regulators (e.g., ERFs, JAZ proteins) and ROS-related pathways. Melatonin and GABA emerge as conserved, non-classical regulators of cold tolerance, consistently associated with ROS scavenging, Ca^2+^ signaling, and metabolic stabilization across species, positioning them as integrative stress-buffering metabolites.

Despite significant progress in elucidating phytohormone-mediated cold stress responses, important knowledge gaps remain, including the following: (i) spatiotemporal dynamics of hormone signaling under CS, particularly tissue and cell-type-specific responses during chilling versus freezing, are poorly resolved; (ii) most studies focus on early vegetative stages, leaving reproductive-stage cold tolerance underexplored; (iii) genotype-dependent hormone–cold interactions in crops remain insufficiently characterized, limiting translational predictability; (iv) interactions between hormone networks and metabolic signaling pathways (e.g., GABA shunt, sugar signaling) are incompletely integrated into cold stress models; and (v) stress memory effects, long-term field-level consequences, and trade-offs between growth, yield, and cold tolerance following hormonal or genetic manipulation are still fragmentary and largely unexplored. Finally, many studies mostly rely on model plants under controlled conditions, which may not fully represent field-level complexity. In addition, the dose-dependent and antagonistic effects of phytohormones can lead to trade-offs between stress tolerance and growth, posing challenges for agricultural applications.

Genome editing technologies, particularly CRISPR/Cas systems, offer powerful opportunities to translate mechanistic insights into crop improvement; however, realistic expectations are essential. Rather than targeting single hormones, future efforts should prioritize conserved regulatory nodes with demonstrated multi-pathway integration, such as *OST1*/SnRK2, ICE1/CBFs, DELLA proteins, JAZ repressors, and key biosynthetic enzymes involved in melatonin (e.g., SNAT) and GABA (e.g., GAD) pathways. Multiplex editing strategies may allow for simultaneous fine-tuning of stress tolerance and growth, but potential pleiotropic effects, developmental penalties, and environment-specific outcomes must be carefully evaluated. Importantly, gene editing should be complemented by systems-level analyses and field validation to ensure agronomic relevance.

Overall, by linking hormone signaling networks with evidence strength, stress type, and actionable genome editing targets, this review provides a decision-oriented framework to guide future research and breeding strategies. Such an integrated approach will be critical for developing climate-resilient crops capable of maintaining productivity under increasingly variable low-temperature environments.

## Figures and Tables

**Table 3:** Integrative framework of phytohormone-mediated cold stress tolerance: key regulatory nodes, phenotypic outcomes, and candidate targets for genetic improvement.

Cold Scenario	Hormone(s)/Metabolites	Shared Regulatory Node(s)	Key Downstream Effectors	Dominant Phenotype	Evidence Base	Candidate Targets
Chilling (0–10 °C)	Auxin	PIN-ARF, AUX/IAA, ROS/Ca^2+^	Cell elongation and photosynthetic genes, ROS scavengers	Seedling establishment, membrane stability, ROS balance	Model + crops	PIN5b, ARF16
Chilling and freezing	ABA	*OST1*/SnRK2, ICE1, PP2Cs	ICE1, COR, CBFs, LEA genes	Stomatal control, osmoprotection, membrane stabilization	Strong (model + crops)	*OST1*, NCED, PP2C, ICE1
Chilling	Ethylene	ERFs (ERF1, ERF13), EIN3/EIL1	ROS detox genes, CBFs modulation	ROS homeostasis, stress acclimation	Moderate	ERF1/ERF13, EIN3
Chilling	Cytokinin	CKX, IPT	Growth-related genes	Growth–stress balance	Model + crops	CKX, IPT
Chilling	Strigolactones	MAX2	Stress-responsive TFs	Shoot architecture, tolerance	Emerging	MAX2
Freezing (<0 °C)	Jasmonic acid	COI1–JAZ, MYC2	ICE–CBF, antioxidant enzymes	Freezing survival, antioxidant defense	Strong (model + crops)	JAZ repressors, COI1
Freezing	Gibberellins	*DELLA*	Growth restraint, CBF activation	Energy conservation, growth suppression, survival	Moderate	*DELLA*
Both	Brassinosteroids	BZR1/BES1	ROS homeostasis	Growth–stress balance	Emerging	BZR1, BRI1
Both	Salicylic acid	NPR1, TGA TFs	Redox signaling	Stress priming, ROS signaling	Moderate	NPR1, ICS1
Both	GABA	GABA shunt, GAD, ALMT, Ca^2+^	Metabolic balance, ROS signaling	Stress signaling modulation	Emerging	GAD, ALMT
Both	Melatonin	SNAT/ASMT, ROS–Ca^2+^	ROS/Ca^2+^ signaling, CBFs	Redox balance, photosystem protection	Model + crops	SNAT, ASMT

Evidence base: Model = Arabidopsis; crops = rice, wheat, maize, tomato, fruit crops.

## Data Availability

No new data were created or analyzed in this study.
